# Philosophical Anatomy

**Published:** 1837-07-01

**Authors:** 


I. Philosophie Anatomique. Par *M. le Chev. Geoffroy Saint Hilaire.*—2 vol. Paris, 1818-1822.

II. Histoire des Anomalies de l'Organization, &c. Par *M. Isodore Geoffroy St. Hilaire.*—3 Vol. Paris, 1832-6.

III. Sketch of the Comparative Anatomy of the Nervous System, with Remarks on its Development in the Human Embryo. By *John Anderson,* M. E. S. London, 1837.

Philosophical Anatomy, or that science whose object is to explain the organization, development, and functions of animals, has hitherto met with very little attention in this country. We seem to have been too stedfastly engaged in pursuing those branches of the profession, which have an immediate and direct application to the improvement of medical and surgical practice, to have spared much time for the investigation of the more abstruse and speculative truths of physiological research. It may be that our Continental brethren have carried the contrary spirit to an extravagant degree; and that, from allowing themselves to be over-occupied with the study of what has been called Transcendental Philosophy, they have neglected the more homely, yet certainly the far more useful, occupation of treating every-day disease. Be this as it may, it is certainly to be regretted that in Britain we have made so little progress in a field of enquiry, which, even if it had no practical advantages to recommend it, ought to engage the attention of every medical man, from the surpassing interest of its objects, and from the grand and impressive truths which it unfolds.

But we may be quite assured that whatever improves our knowledge of any branch of anatomy and physiology, will ultimately improve our knowledge of disease, and of the means best fitted to relieve it.

It is a great error to suppose, that *he* makes the best practitioner, who devotes his time and study exclusively to the mere minutiæ of medical or surgical experience, and whose thoughts are constantly occupied with reports of cases, effects of remedies, and the innumerable opinions of different writers.

It was not by treading in such a narrow path, that Hunter achieved his glorious renown, or that Haller, Morgagni, or Monro have shed a lustre over the profession which they adorned; and, in short, all the great ornaments of medicine, in bye-gone as well as in the present time, have been and are those men, who have most deeply and most sedulously explored those very branches of knowledge, which we, perhaps unfortunately, denominate the accessory departments of professional education. The names of Scarpa, Bichat, Magendie, Charles Bell, Brodie, not to enumerate a host of others, will be admitted by every one, as affording the best proof which we could adduce of the truth of our position, that philosophical researches are not unfavourable to the acquisition of the greatest practical skill.

Of such studies there is none which will contribute more essentially to improve and exalt the character of the profession, than that of anatomy and of physiology, in their widest and most comprehensive range. The full extent and marvellous interest of these sciences have never been duly appreciated either in our schools, or by our writers. We have been too much given to limit our attention to the mere pursuit of a knowledge of human anatomy and human physiology; as if these alone were sufficient to disclose the admirable truths of life and of organization. This has been a great and most prevalent error. To understand aright the philosophy of living structures and of the functions which they are destined to perform, it is absolutely necessary to study them, not in one animal only, but in all the classes and orders of the animal kingdom.

Hence the first-rate importance of comparative anatomy and of comparative physiology, to illustrate the anatomy and physiology of man. Within the last few years, it is most gratifying to have observed the growing fondness for the former sciences, which has been displaying itself in this country. Already there are several established lecturers on comparative anatomy in London. The London University has the merit of having set the example; and the Royal College of Surgeons, by their recent institution of an annual course at their Hall, has most properly recognized the importance of the study.

The bulk of our readers have only to call to mind the character and style of the lectures on physiology, which they were accustomed to hear delivered during their studies, to be satisfied how feebly and how imperfectly its most sublime truths were taught. Perhaps during the whole period of a six-months' course, not above half-a-dozen of passing allusions were ever made to the structure and development of the lower animals.

The professor was quite satisfied with tediously narrating the most obvious phenomena, which such and such organs in man exhibit during life. For example, he would occupy a whole or perhaps several lectures in telling his auditors that the liver secretes the bile, that the bile is a bitter acrid substance, that it assists in the assimilation of the food, and promotes the peristaltic action of the bowels, and other such-like meagre details. All this information is very good, and very proper to be well understood by the student, to prepare him for the more practical details of nosology. But his instruction ought not to cease here; he should be taught to enquire what is the structure and development of the organ in the different tribes of animals, what changes it undergoes according to their habits and modes of life, what influence it appears to exert on other organs and other structures of the body, and in what manner its absence in certain classes seems to be compensated for.

The case, we have now put, would have been much more forcible, had we selected the stomach in place of the liver, as the subject of investigation; but this matters little, since both organs are intimately connected with each other, and are engaged in the performance of one function—the digestion of the food and its conversion into nutritive chyle. This primary and most essential of all the operations of living bodies, cannot, in the nature of things, be rightly understood, by studying its phenomena in one animal only, or in one tribe of animals. Here has been the great mistake in the teaching of physiology. The function of digestion has been described only in reference to man, with perhaps a few passing remarks on its peculiarities in some of the more common herbivorous and carnivorous animals. But no comprehensive view of this wonderful attribute of life in the different classes of animals, from the most complicated to the most simple, has ever been presented to the medical student. The lecturer on physiology might have received a useful hint, had he only attended to the mode of investigation, which is followed in the pursuit of other sciences. The chemist is not satisfied with discovering or with describing one only of the properties of a substance. He tries it with all sorts of tests, submits it to a multitude of examinations, and compares its qualities with those of other substances of a similar character, before he expects to arrive at a correct knowledge of its qualities. The same general course of minute and comparative research must be introduced into the teaching of physiology, before we can hope to attain to any philosophical exactitude in our acquaintance with the mysteries of life. Every part, organ, and function of the body should be studied and explored in the different tribes of animals; their modifications, changes and varieties should be brought together and compared with each other; and then, but not till then, shall we be prepared to deduce any rational conclusions as to their uses and their powers.

As it is ever a pleasing part of a Reviewer's duty to bestow praise, we gladly avail ourselves of the present opportunity to express our very high esteem for the great abilities and unwearied assiduity of Dr. Grant, the distinguished Professor of Comparative Anatomy in the University of London. His course of lectures, which adorned the pages of the Lancet two or three years ago, has contributed most essentially to the diffusion of high and philosophical principles in the study of physiology; and we trust that Mr. Wakley, with his accustomed zeal and enterprize, may be induced ere long to publish Dr. Grant's lectures on this delightful branch of medical study. There is certainly not a man in this country better fitted to do justice to the subject. His intimate acquaintance with human as well as with comparative anatomy, the variety and accuracy of his scientific attainments, the philosophical cast of his mind, and, withal, the impressive eloquence of his language admirably qualify him as a teacher of physiology.

We have been much disappointed in finding that his services have been so little called into requisition in the composition of one of the most meritorious works of the present day—we allude to the Cyclopædia of Anatomy. Dr. Todd will do well not only for the credit, but also for the permanent success of his book, to entrust many of the physiological articles to such a man as Dr. Grant. It was only the other day that, in making reference to one of the very first articles in the first number professing to give an account of a most important function of life, we had occasion to lament its great and lamentable imperfections. Page after page is occupied with details of literary history, commencing as usual with Hippocrates and Aristotle, and most wearisomely mentioning name after name down to the present time—a species of information more curious than useful; but, throughout the whole article, there is scarcely even a reference made to the state and development of the organs in any of the lower classes of animals; and yet without such knowledge, it is impossible to understand aright the functions which these organs are designed to perform.

We may be quite assured that it is only the comparative anatomist that can ever hope to be the scientific physiologist. The study of human anatomy and physiology alone may make the clever operator in surgery, and may even be deemed sufficient for the purposes of the practical physician in the ordinary routine of his avocations; but it can never suffice for him, who has any taste for the philosophy of his profession. As well might the geologist hope to be satisfied with a mere knowledge of mineralogical details, and with having a partial acquaintance with a certain district of country. The great and wonderful truths of geology could never have been established without a comprehensive review of the structure of the earth in different regions, and a minute examination not only of the mere minerals which enter into its composition, but of a multitude of accessory objects and of accessory phenomena, which at one time were deemed quite alien to such enquiries.

Every one must have heard of the glorious results of Cuvier's application of comparative anatomy to illustrate and confirm the grand discoveries of this science; and he indeed would be deemed, in the present day, little short of a madman, who might presume to expatiate on any theory of the earth's formation, without having a strict regard to the obvious conclusions, which are presented to our minds by the study of fossil osteology. Equally vain will be the attempt of the anatomist to explore the paths of physiology, if he limits his researches to one particular object, or to one set of objects in the animated world. Comparison and analogy are two of the most powerful implements of the mind, in any effort to arrive at truth. In the study of natural history, it is alike delightful and instructive to call them to our aid; and, under their guidance to follow out, step by step, the grand chain of organic existence, tracing each part from its simplest form up to its most complex development; to observe the striking, yet unlooked for, resemblances between the same organs in different animals and in different classes of animals; to mark the all-admirable skill, with which Nature has adapted and accommodated each being to its necessities, wants and desires; and thus to derive fresh and fresh proofs of the unity of that design, which has formed and fashioned every part of creation. Such is the noble end, and such are some of the sublime results which the study of comparative anatomy and physiology will suggest to the generous mind.

There is another and kindred department of the science to which we have not yet alluded, but which is intimately connected with the object of the present enquiry, and merits an equal share of our attention—we allude to the study of Embryology, or that department, which professes to trace the successive steps in the development and evolution of an animal (more particularly of man), from the early stage of intra-uterine existence, until it arrives at its mature and perfect formation. This is a branch of physiology, almost quite unknown in this country, although during the last twenty years it has been investigated with great zeal and success on the Continent.

It is to the labours of the German and French anatomists, more especially to those of Meckel, and Tiedemann, of Geoffroy St. Hilaire and Serres, that we are indebted for almost all that we know on this interesting subject. Their researches have made known to us the wonderful truth, that the intra-uterine being passes through a series of successive changes or phases of existence, beginning with the most simple state and gradually advancing to the more complicated and perfect. They have shewn us, that the human embryo, at the earliest period of its evolution, is analogous to some of the simplest members of the zoophytic class; being nothing save a mere vesicle or globule, filled with a glairy fluid, and exhibiting no appearances of variety of parts; and that gradually organ after organ, and system after system are developed and more and more complexly formed; each successive change representing as it were, the structure and formation of an animal, higher and higher in the scale of organization. We shall attempt in the sequel to point out and illustrate many proofs of this law. For example, when we come to treat of particulars, we shall endeavour to shew that the nervous system in the very young embryo is nearly analogous to that in the more simple of the invertebrata; that it next assumes an arrangement, such as we find in the insect and mollusk tribes; and that successively, as it becomes more and more complex, it may be aptly compared to its organization in fishes, reptiles, birds and lower mammals, until at length it arrives at its perfect development, such as exists only in the human species.

The progress, that has been already made in this most curious field of physiological enquiry, encourages us to hope that, ere long, some work may be published in the English language, presenting a comprehensive summary of all that is already known of embryological anatomy. We are not aware that even an attempt at such an undertaking has yet been made. The works of Mr. Anderson and of Mr. Solly on the Brain are creditable; but they embrace only a very limited portion of the subject. We should be glad to hear that such an anatomist as Dr. Grant, or Mr. Owen the professor of comparative anatomy to the London College of Surgeons, or Dr. Knox of Edinburgh had directed their attention to an enterprise, which is well worthy of their best exertions. And let it not be supposed that the study of such philosophical enquiries is attended with no direct benefit to medical science.

One of the most interesting proofs of their great importance is afforded us by the consideration, that since embryology has been studied with scientific precision, much light has been thrown on the hitherto mysterious subject of Monstrosities and congenital malformations. It has, for example, been most clearly proved that a considerable proportion of these anomalies may be very easily and satisfactorily explained; and we may add that already a decided improvement has been introduced in the treatment of many of them. It would require a much wider field, and a far more protracted study than we can at present afford, to treat this subject in all its details.

The curious reader will find a large mass of most useful information in the three volumes of the younger St. Hilaire. This work is certainly a very meritorious production, and reflects great credit on the zeal, industry and talents of the author. We have perused it very carefully, and have endeavoured to compress as much of its interesting matter in the following pages, as the scope of the task, which we have assigned to ourselves, would permit.

The object of this task we may briefly state to be, an endeavour to point out and to illustrate a few of the most interesting discoveries which have flown from the study of Embryology, and to shew the application, which has been made of these, to explain the occurrence of many of the structural anomalies or malformations, known under the name of Monstrosities.

In the course of the enquiry, we shall have frequent occasion to refer to the facts of comparative anatomy, and to remark the intimate analogy which may be traced between the organization of the fœtus, at different periods of its existence, and that of the different classes of animals.

Two theories have been proposed to explain the mode of development of the embryotic animal. According to the one—that so eloquently enforced by Haller, and which has, until of late years, been very generally adopted by anatomists—the great systems or divisions of the body, such as the vascular, the nervous, &c. are supposed to be formed from the centre towards the circumference; the heart, brain and spinal cord being, it is imagined, first generated, and the blood-vessels and nerves radiating from them, as from a middle point, to all parts of the surface. It was long, indeed, a question of dispute in the schools, whether the heart, or the cerebro-spinal axis, was the part first formed; but, whichever of these opinions was adopted, all physiologists, without exception, seemed to believe that these two parts made their appearance before either the blood-vessels or nerves. Every reader must have heard of the 'punctum saliens,' the embryotic heart, being usually described as the earliest sign of a circulatory apparatus; and, even in the present day, perhaps the majority of medical men are not aware that the truth of this opinion has been ever called in question.

The rival doctrine maintains the very opposite conclusions. According to its precepts, the development of the animal body is from the surface of its two lateral halves towards the mesial line or centre. To recur to the case of the vascular and nervous systems, M. Serres (one of the most accomplished anatomists of the present day, and long distinguished among the French anatomists by the boldness and ingenuity of his speculations) asserts, and his assertion has been confirmed by the researches of many other enquirers, that the blood-vessels and nerves are developed primarily in each lateral half of the young embryo, and that they gradually converge towards the mesial line of the body, until they at length finally coalesce, and thus give origin, as it were, to the heart and its large trunks on the one hand, and to the brain and spinal marrow on the other.* Such is the doctrine which has been denominated the 'Centripetal or Eccentric Theory of Development,'† in opposition to the more ancient one, to which the appellation of the 'Centrifugal Theory' has been given. These terms are sufficiently expressive; and we shall therefore, for brevity's sake, make use of them in the following pages.

Our author—and he must be considered as no mean authority on all questions connected with embryogeny—is a most zealous supporter of M. Serres' views. His work indeed is based throughout on the centripetal theory; and affords, it must be admitted, a host of very strong proofs in favour of its correctness. It is to be regretted that M. St. Hilaire has not given a condensed and succinct view of these proofs by themselves. They are scattered over almost every chapter of the book; and it has given us no small trouble to pick them out and bring them together within a short space. We shall first allude to the arguments which may be derived from the development of some parts of the osseous system in favour of the centripetal theory, and we shall select for our purpose those parts of this system, which are situated in the line of the mesial plane.

---

* What adds great probability to this doctrine is the fact, that the heart has been found wanting in some monsters, while the blood-vessels were present; but the reverse of this condition has never been detected. The same remark may be made of the cerebro-spinal axis and of the nerves—the former may be absent, but the latter are always present.

† Some German anatomists have, in compliment to its distinguished proposer, called it the Lex Serriana.

The cranium is believed to afford most convincing illustrations of the centripetal law of formation. The eight bones of the cranium may be conveniently arranged into those which are situated in the mesial line and are single in the adult body, such as the os frontis, os occipitis, os ethmoides and os sphenoides; and into those which are situated on each side of this line, and which are, it is well-known, double—viz. the parietal and temporal bones. In the young fœtus, the single bones are, each of them, composed of two lateral halves, which may be said to correspond to the two parietal and the two temporal bones. Every young anatomist is aware that the os frontis not unfrequently exhibits a mesial suture—a proof of the bone having at first consisted of two lateral parts, which in most crania coalesce so intimately at an early period of life, that the line of fissure is effaced. It is also a well-known fact, that the fœtal os occipitis consists of four segments; one superior and posterior, another inferior or basilar, and the other two lateral, being situated on each side of the foramen magnum. In the very young embryo however the number of these pieces is greater; for the posterior and the basilar portions are then found each to consist of two lateral symmetrical halves not yet united. They subsequently coalesce along the mesial line, and all traces of their former separation are effaced. The line of the mesial coalescence of the posterior segment is where the falx cerebri and cerebelli is attached. The ethmoid and sphenoid bones also are composed primarily of two lateral parts, separated from each other along the mesial line.*

It thus appears that the line of the sagittal suture extended forwards and backwards around the whole cranium in a vertical direction, separating one lateral half of the skull-cap from the other. As fœtal life advances, these two lateral halves have a tendency to meet together and to coalesce; and this process takes place in every point except between the two parietal bones, which, for wise purposes, are kept apart from each other, until after the birth of the child.

It will be observed that, we are not speaking at present of the process of ossification in the separate bones. This is quite another subject of enquiry. All that we wish to prove is, that there are two analogous formations in each lateral half of the cranium; that there are two frontal, two occipital, two ethmoid, and two sphenoid, as well as two parietal and two temporal bones; and that all these laterally situated bones are developed more or less apart from each other at first, and have in the progress of their development a tendency to meet together and to coalesce. If our limits permitted, we might allude to the development of many of the facial bones to illustrate

---

* The exact accuracy of these statements has been ascertained by the researches of Meckel, the elder St. Hilaire, Serres, &c. on the anatomy of the young fœtus; and it has been most beautifully confirmed by the discoveries of comparative anatomy and by some interesting phenomena of teratology. For example, in certain cases of cephalic monstrosity, the brain or some portion of it has been found protruding, not only at the bregmata and between the two parietal bones, but also between the lateral portions of the frontal and occipital bones, and even right through the centre of the ethmoid and sphenoid bones, in the situation of the nasal lamella of the former and of the sella turcica of the latter.

the same general law of lateral duality, and of mesial union. The ossa nasi and the superior maxillæ might be aptly quoted for this purpose; but perhaps the inferior maxilla affords a still more interesting illustration. This bone consists at first most truly and essentially of two lateral halves, altogether analogous, or rather similar to each other: these two halves afterwards meeting together at the symphysis menti in the mesial line, and there coalescing by a most intimate union. There cannot be a more conclusive illustration of the "Lex Serriana," or doctrine of centripetal formation, than is afforded by the progressive development of this bone.

Let us now direct our attention to some of the bones of the trunk. The vertebral column consists, it is well known, of a multitude bones, all bearing a general analogy and resemblance to each other, and all truly symmetrical—their two lateral halves being strictly alike. Now in the very young embryo, these two halves of the vertebræ are found to be formed apart from each other; each half having its rudimentary body and lateral processes. The spinous processes of the vertebræ are the parts latest developed; and hence, for a considerable period of intra-uterine life before they are formed, the spinal canal is open posteriorly, along its whole extent from the cranium to the extremity of the sacrum. It sometimes happens that, at particular parts, this open state of the spine continues at the period of birth; and then we have presented the malformation, which has been called "spina bifida." The etiology therefore of this affection is merely a retardation to, or arrest of, the normal process of development—the vertebræ remaining, in some respects, in their embryotic state, instead of meeting together and coalescing behind as they usually do. As every student of medicine is acquainted with the history of "spina bifida," we need not at present say more on this subject. But there are probably few of our readers who are aware that the bodies of the vertebræ, as well as their spinous processes, sometimes exhibit this malformation. In certain monsters, the two lateral halves of some vertebræ, more especially of the dorsal, are actually disunited along their mesial line; and then either the spinal cord may protrude between the fissured bones into the posterior mediastinum, or a portion of the lungs into the vertebral canal. It was, by the study of such structural deviations as these, that M. Serres was first led to deduce his glorious discovery—the law of centripetal formation.

The sternum, a single bone situated in the mesial plane, and not exhibiting any traces of "lateral duality," was for some time supposed to argue against the universality of the "Lex Serriana."

But subsequent researches have shewn the incorrectness of this supposition. The sternum (or rather the several bones which compose it), is actually found in the very young embryo to be divided, by a longitudinal or vertical line of separation, into symmetrical portions. This cleft state of the bone continues to exist in some rare monstrosities; and the fissure is sometimes so wide, as even to permit the heart to be protruded outwardly through it.

It is thus that the study of Teratology, or the science of monstrosities, so beautifully illustrates the most important doctrines of Embryogeny.

From the sternum we might pass on to the consideration of the pelvis, and adduce, from the history of its development, fresh proofs in support of the centripetal theory. But these must in truth be too obvious to every reader, to require any elucidation on our part.*

Leaving, therefore, the osseous system, we shall now briefly direct our readers' attention to the development of some of the soft parts of the body, as illustrative of the centripetal law. Let us take, for our first example, the anatomy of the abdominal parietes.

The linea alba is, all know, situated in the mesial line anteriorly, as the vertebral column is situated posteriorly. We have already shewn that this column is, in its primitive condition, cleft along its longitudinal axis. Equally so is the linea alba.

The abdominal muscles of each side are, in the young fœtus, separated by a fissure from the sternum to the pubis. As yet there is no linea alba; for, in truth, this line is subsequently formed by the meeting of the tendinous expansions of the muscles from each side. Often, indeed, this junction remains imperfect at the period of birth; and then some portion of the abdominal viscera is protruded, giving rise to a congenital ventral hernia.

The external organs of generation afford equally striking proofs of a primitive lateral duality, and of a subsequent "mesial coalescence." In a succeeding part of this article, when we come to treat of the curious subject of Hermaphrodism, we shall describe more minutely some of the more interesting facts, connected with the development of the generative apparatus. At present, therefore, we shall merely allude to those, which more directly bear upon the point under consideration.

The corpora cavernosa of the penis and those of the clitoris are, when first developed, separated from each other in the mesial line of their septa; and, at this period of embryotic life, the urethra also is found to consist of two lateral unclosed halves. The corpora cavernosa, as well as these halves of the urethra, gradually approximate to each other; and ultimately they coalesce firmly together, leaving no vestiges of their primitive disjunction. But if, from any cause, the process of normal development be interrupted, the parts may retain, at birth, the condition of their early formation. In some rare cases, it is well known that the child is born with the penis cleft along a part or the whole of its length; and in others (an anomaly which is much more frequent than the preceding), the corpora cavernosa have united, but the canal of the urethra remains open, as if it had been slit along its inferior surface. To this latter malformation, the name of "Hypospadias," has been given. The proper explanation of this affection will be

---

* As strictly appertaining to the subject under review, we are induced to extract the following passage from a very able descriptive memoir on the amphibia by Mr. Bell, published in the Cyclopædia of Anatomy.

"On examining the general texture of the bones in the amphibia, there is an obvious advance towards the firm calcareous substance of those of the higher vertebrated animals, when compared with the bones of fishes. The cranial bones, though they retain to a certain extent the character of those of fishes in the permanent disunion of the different centres of ossification, yet do not overlap each other as in that class; but, on the contrary, remain with their margins at least in contact, and in many cases actually united, though not by true sutures. The elements, of which the cranium is essentially composed, and which in a higher grade of organization are consolidated, are here still exhibited as distinct pieces—*a state of things which is strikingly imitated in the progress of the development of these parts in the highest classes during their growth.*"

understood from what we have now stated. It is not, as some authors have most incorrectly asserted, the result of disease, but merely and altogether of the arrested or impeded development of the organ.

The scrotum is another part, which is double in the very young embryo. The two bursæ are formed separately, and afterwards coalesce along the line of the raphé. Even the urinary bladder itself seems to be formed in the same manner; but as this point has not been so accurately ascertained, we shall pass on to the consideration of the development of the uterus. It deserves however to be remarked, that in those cases of extreme malformation, in which the urinary bladder is protruded through the unclosed symphysis pubis (its anterior being often awanting at the same time), the fundus of the viscus usually exhibits a bilobed appearance, and the other parts of the genito-urinary apparatus are almost always more or less defective simultaneously. Thus the anomalies of Hypospadias and of fissured scrotum and perineum are very common complications of the "Exstrophia" of the urinary bladder.

The truth of this remark is, indeed, of very general application. It is rare to meet with any great structural deviation of one part in a new-born child, unassociated with other anomalies or deviations; and no doubt for this very satisfactory reason, that the cause—be that what it may—which has induced an abnormal development of one part, must necessarily act upon other parts of the body at the same time. It is in this manner that we are to account for the very frequent co-existence of cleft lip, of fissure of the palate, of umbilical hernia, and so forth, in infants affected with spina bifida. All these malformations are, it will be observed, attributable to the same general cause—namely, the imperfect mesial junction of the two originally separated lateral halves of the body.

Every anatomist is aware that there are numerous examples on record of what has been called "double uterus."

This anomaly consists in the viscus being more or less completely cleft, in a vertical direction, along its mesial plane. In some cases, this fissure is very complete, extending from the fundus to the cervix; but more frequently the fissure is limited, and the uterus then presents the appearance of having a double fundus. [We have recorded a good many examples of double uterus in an article, which our readers will find in the Foreign Periscope of the number for January.] In other cases, the uterus does not exhibit any unusual appearance outwardly; but on being cut open, it is found divided into two compartments, a right and a left one, by an intervening septum; just in the same manner as the heart is divided into right and left cavities by its septum. When such a septum exists, we believe that it is always situated in the mesial line, extending from the anterior to the posterior surface, and never in a transverse direction from side to side. The bilocular uterus may therefore be considered as an inferior or less complete degree of the bilobed uterus; and the explanation of both anomalies is the same. In the young fœtus the uterus is found to consist of two lateral parts, not yet agglutinated to each other. As the coalescence and fusion of these two parts take place, the septum is gradually absorbed, in the same manner and probably according to the same law, which determines the absorption of the membrana pupillaris of the iris.

If necessary, we might allude to the embryotic development of other viscera, situated in the mesial line of the body, to find fresh proofs of the operation of the centripetal law.* But we prefer to appeal to other parts and other structures, whose anomalies are better known, and have been more attentively investigated. None are better suited to our purpose than those of the lips, especially of the upper one, and of the palate. Median fissure of the upper lip (one of the varieties of harelip), of the soft and hard palates, and of the velum pendulum and uvula, is a malformation of not unfrequent occurrence.

Before the time of M. Serres, no satisfactory explanation could be assigned of the origin of these structural deviations. Some anatomists thought that they were the result of some disease or another affecting the fœtus; while others very wisely conjectured that the tongue must, somehow or other, have forced a passage through the palate, while it was forming! This may be taken as a specimen of the idle and very foolish ideas, which prevailed on the subject of monstrosities within even the last half century. The discovery of the great law of centripetal development has not only dissipated all these absurdities, but has enabled us to explain satisfactorily, and in a manner worthy of such a science as that of embryology, this and most other congenital deviations of structure. The fissure of the upper lip and of the palate is now most indisputably known to be the result not of any disease or injury, but simply of an arrest or stoppage of the development of the part, in its progress to mature formation.

In every young fœtus without exception these parts are fissured, just as we find them to be in cases of harelip and of cleft palate. It is the natural state of the parts, while they are *forming;* and it is only at an ulterior period of intra-uterine existence that the lateral halves of the lip and of the palate, are united, and, as it were, fused or melted into each other. Now if, from any cause, nature is stopped or impeded in its operation of developing a part, it will necessarily follow, that, at the period of birth, that part will continue to exhibit the appearance which it had when the stoppage or impediment of the process took place; while the other parts of the body, which had encountered no such arrest, will have advanced to the normal completion of their development. This is the key of solution to many of the problems of Teratology; the Ariadne-thread, which will guide the anatomical philosopher through its apparently most intricate and mysterious mazes.

It is, therefore, of primary importance that the student should ever bear in mind the memorable fact that, in every class of animals, all the parts and organs, situated in the mesial line of the body, consisted primarily of two symmetrical analogous lateral halves; that these halves are developed separately from each other; that they have a gradually increasing tendency

---

* The heart, not being situated in the mesial line, might at first be supposed not to be subject to the operation of the centripetal development.

But the investigations of Serres and others have most distinctly proved that this most important organ is situated in the young embryo in the mesial line, and that its lateral halves are developed apart from each other, and afterwards coalesce together.

The fissure of the apex of the heart—an occasional anomaly, and which is the normal state at a certain period of fœtal life—is a vestige of this coalescence having been interrupted.

to meet and coalesce in the mesial line; and that most of the congenital malformations of these parts are attributable to an interruption of this mesial union and coalescence. Such is the sum and substance of M. Serres' great discovery—the law of Centripetal or Excentric development.

We have already said that the heart, during the successive periods of its formation, appears to obey this general law. As the development of this organ is of the highest interest, we shall describe at some length the most important facts, which have been ascertained respecting it in the chick, as well as in the fœtus of man and of other mammiferous animals.

We shall be led at the same time to compare the successive phases or stages of development of this organ in the higher animals, with its permanent forms or conditions in those lower in the scale of organization; and thus to perceive the truth of that marvellous harmony and unity of design, which pervades the whole scale of animated nature.

In the chick, 30, or 40 hours after the commencement of incubation, the heart appears as an open sac. This sac gradually closes; soon afterwards it begins to exhibit a bulging out or projection at three different points of its surface. These three protuberances are separated, imperfectly indeed, from each other by intermediate contractions or puckerings. They correspond to, and are in truth the rudimentary formations of, the future left ventricle, the left auricle, and the bulb of the aorta. The heart is therefore, at this period of embryotic existence, of a very simple conformation; consisting of but one cavity, and thereby resembling the heart in some of the lower tribes of animals. As yet there is no distinct septum, or any appearance of a valve between the auricle and ventricle. While this septum is forming, the right ventricle begins to appear as another protuberance or bulging out at the side of the left ventricle; and soon afterwards the development of the right auricle follows. The two auricles at first form but one cavity. The partition between them is developed subsequently, and seems to be generated by the gradual approximation of a fold of the walls of the auricle descending from above and by another ascending from below, until these coalesce, except at one point where the foramen ovale is situated.

It has not yet been distinctly ascertained whether the two ventricles, like the two auricles, form at first but one common cavity. This however at least is certain, that towards the end of the second month of intra-uterine life there is, in the human fœtus, an aperture through the base of the septum ventriculorum, permitting a free communication between the two sides; and moreover that the aorta, at this period, springs directly from over this point. The pulmonary artery as yet appears to be scarcely developed.*

---

* The following brief description of the development of the heart is taken from Mr. Solly's Work on the Brain.

"The first appearance of the heart in the human embryo is that of a mere pulsating vessel without any division into cavities or thickening of its walls; an arrangement which in all its simplicity is met with as the sufficient instrument for effecting the circulation in the perfect insect.

The next step consists in the gradual dilatation of this tube into a sac, previously to its division into four cavities; and this corresponds with the single heart of the fish, consisting merely of an auricle for the reception of the blood, and a ventricle for its propulsion.

The study of the various phases in the formation of the heart, now alluded to, is especially interesting, when we contrast them with the conditions of the central circulatory apparatus in the different classes of animals, and when to these we connect some of the phenomena exhibited to our view in various monsters. We find that there is an intimate analogy between these successive phases on the one hand, and the normal state of the heart in the lower tribes of animals and also the anomalies which are found in monsters on the other hand.

To illustrate this very curious subject, we shall first briefly allude to the comparative anatomy of the heart, and endeavour to point out the resemblance between the structure of the organ in some of the classes of animals, and the successive conditions of it in the human fœtus.

As a general remark, it may be stated that the organization of this viscus is found to exhibit a gradually increasing complexity of structure, from the simpler animals in which we perceive only its rudimentary condition, up to the mammalia and to man, in whom it reaches its maturity of development.

In the zoophytes and entozoa, there are no traces of any heart. In insects, the dorsal canal, which is closed at each extremity, may perhaps be regarded as the earliest rudiment of a heart. In the tribe of the arachnidæ, comprehending the spiders, scorpions and such like animals, this dorsal canal appears to have acquired the distinctive characters of a heart; as it may not only be seen to pulsate through their bodies, but also because we find vessels proceeding from it and going to different parts of the body.

The dorsal vessel, in some of the vermes, exhibits several bulgings or swellings, which have been considered as analogous to so many hearts: but this supposition is of doubtful accuracy. Among the crustacea, the central circulating organ of the branchiopoda is somewhat similar to that of insects, being of an elongated form, and more like a large artery than a distinct heart; but in the decapoda (the section including the crabs, lobsters, &c.), it is rounded and has a more decidedly heart-like character. In both of these groupes, the organ has but one simple cavity, which receives the veins of the body, and from which issue the arteries of the branchiæ. The vascular apparatus therefore in these animals corresponds to the right or pulmonic heart of the mammalia.

In the mollusca, the heart is found to exhibit a more complex structure. In the gasteropodous and pteropodous sections, it consists of an aortic ventricle and of a pulmonary or rather a branchial auricle—in the acephala, of an aortic ventricle, and of two pulmonary auricles—in the brachiopoda, of two aortic ventricles which are separate from each other, and act the part of auricles at the same time—and in the cephalopoda, of three ventricles, two pulmonary and one aortic, all detached from each other and provided with auricles.

---

As the development advances, a second ventricle is added to the first on the right side of it, separated from the left by a septum, which is so imperfect that the aorta communicates with both cavities; and the very same arrangement is found to exist in the adult crocodile.

While the septum is being formed in the interior, a notch appears on the exterior, which, extending from the apex to the base, divides the heart in exactly the same manner as it is met with in the dugong."

When from the mollusca we ascend in the scale to the class of fishes, and examine their central circulatory apparatus, we discover that there is a considerable resemblance between it, and that of the Gasteropoda already described. The heart consists of but one auricle and of one ventricle; the former receiving the veins of the body, the latter sending out the branchial artery.

In reptiles, there is one ventricle, and either one or two auricles; but the circulatory apparatus in these animals differs very essentially from that in fishes and in the mollusca, inasmuch as not only the veins of the body, but those also of the lungs terminate together in the auricle, and as the ventricle gives out two sets of arteries, of which one is destined for the general, and the other for the pulmonary circulation.

The heart in birds and in mammiferous animals possesses all the characters and general arrangements of the human heart.

This brief sketch of the comparative anatomy of the heart may probably suffice to indicate to our readers the very striking resemblances which may be traced between the progressive developments of the viscus, during its evolution in the embryo, and some of its congenital malformations on the one hand, and the normal condition of its structure in the inferior tribes of animals on the other hand. We have already seen that the heart, when first discoverable in the fœtus, appears to consist of but one or two, imperfectly separated, cavities; and in some cases of extreme monstrosity the heart has been found at this *Zero* of its development. Now it will be remarked that this state is strictly analogous and corresponding to the normal condition of the organ in some of the lower animals, as in the insecta and certain crustacea.

The same train of comparative investigation may be applied to the progressive changes which the heart undergoes during its embryotic evolution, from its earliest appearance to its mature and perfect formation.

The younger the fœtus, the more simple is its structure; and it progressively becomes more and more complicated in its organization, just in the same manner that it is known to acquire a gradually increasing complexity, as we ascend from the lower to the higher classes of animals. It is by pursuing this most curious subject of investigation, that we are enabled to give a rational interpretation of such congenital malformations, as the existence of only one auricle and one ventricle, or of a free communication between the right and left sides of the heart, or of the aorta arising from both ventricles, and such like irregularities of structure.

We cannot dismiss the subject of the changes in the central circulatory apparatus, (not only in the different classes of animals, but also in the same animal during the progressive periods of its evolution until it reaches its mature development,) without directing our reader's attention to the very wonderful metamorphosis which this apparatus, as well as other parts of the body, undergoes in the young frog, when passing from the state of tadpole to that of a perfect animal. The tadpole or young frog is in most respects strictly and essentially a fish. It has no developed anterior or posterior extremities; the coccygeal vertebræ are numerous and lengthened out so as to form a tail by means of which the animal propels itself, in genuine piscine manner, through the water; the blood is purified by means of gills exposed to the water, and not of lungs which admit air; and many parts of the osseous system, especially the bones of the cranium, exhibits most of the characters of those of fishes. While the marvellous change from a branchial or aquatic to a pulmonic or air-respiring animal is going on, the limbs become gradually developed, passing through a rudimentary form beneath the integuments, from which they do not emerge until they have acquired considerable size and a very defined figure. When these members are fairly formed, the tail is slowly removed by *interstitial absorption*,* and does not suddenly fall off, as some writers have most erroneously maintained, and the animal is thus gradually fitted for its new mode of life.

The changes in the circulatory and respiratory organs deserve to be explained at somewhat greater length. We shall avail ourselves of the description which Mr. Bell has given of it, in the Cyclopædia of Anatomy.

" Beginning," says Mr. Bell, in the article already quoted, " life with all the essential characters of fishes, even in the functions of circulation and respiration, possessing the single branchial heart of that class, how wonderful and beautiful are the changes which these organs undergo, as the branchiæ become obliterated to give place to pulmonic cavities, and the heart at the same time assumes the compound character and form of a systemic and pulmonic heart, in accordance with the change in the respiratory organs. The heart consists at first of a systemic auricle, which receives the whole of the blood from the system, and of a ventricle which propels it into the branchial arteries. It returns by the branchial veins, which, as in fishes, concur to form an aorta without an intervening ventricle. From the last branchial artery on each side is given off a branch which goes to the rudimentary pulmonic sac, and which ultimately forms the trunk of the pulmonary artery. The most interesting change is that, by which the continuous branches of what were originally the branchial arteries combine to form the two trunks of the aorta.† The two veins, which return the blood from the rudimentary air-sacs gradually enlarge, as these cavities become more important, and assume the character of lungs. These by degrees become, as it were, distended at their point of union with the heart, and ultimately form the second auricle. The branchial arteries become gradually more and more contracted, until at length they are quite obliterated. In the adult condition of the animal, when it has now become an air-respiring creature, the heart consists of a single ventricle and of two auricles. The little

---

* It is of great importance for the student of embryological anatomy to consider most attentively this topic of the gradual absorption of a part, while an animal is passing from a less to a more perfect form of existence; as it constitutes an essential element in the doctrine of excentric or centripetal development. That certain parts, present at one period of fœtal existence, are subsequently absorbed, is most indisputably proved by the disappearance of the membrana pupillaris of the eye.

The removal of the tail in the young frog may be adduced as an operation altogether analagous. It is by an extension of this same principle, that M. Serres explains one of the steps in the development of certain mesial hollow organs, such as the womb, &c.—which he supposes to consist at first of two separate cavities, gradually coalescing, until they constitute but a single unilocular organ, the central septum having been removed by interstitial absorption.

† Those who wish to make themselves better acquainted with the details of this most interesting subject will find much valuable assistance in the " Tabular View of the Circulation in vertebrated Animals," by M. St. Ange. An English translation of this work has been published by Mr. Jones.

pulmonic auricle receives the aerated blood from the lungs by means of the pulmonary veins. The systemic auricle at the same time receives the impure blood from the system by the venæ cavæ. The blood from the two auricles is sent together into the single ventricle, where it becomes mixed, and this mingled arterial and venous blood is propelled by the same impulse, partly into the pulmonary arteries to be more perfectly purified, and the remainder through the aorta to the different organs of the body."

We trust that our readers will be pleased with the lengthened details which we have given respecting the development of the heart. The subject is one of great interest, and cannot fail to engage the attention of every medical man, whose thoughts can go beyond the pleasure of pocketing a fee, or the bore of writing a prescription.

We have selected the heart as our primary illustration of that most important discovery in embryological science—that the bodies of the more perfect animals undergo, during their development, a series of progressive changes from a more simple to a gradually increasing more complex formation, and that these consecutive changes of structure are strictly analogous to the normal condition of the organs in the lower tribes of animals, because the comparative anatomy and physiology of the circulatory apparatus, is probably very generally known to the mass of our readers. Other organs however furnish perhaps even more conclusive proofs of this great law. The testicles may be aptly quoted for our purpose. It is well known that in a number of animals the testicles never descend through the abdominal rings, but remain during life in the loins. It is also known that during the first seven or eight months of intra-uterine life of the human fœtus, the testicles are situated immediately beneath the kidneys, and that then they gradually descend to the groins and subsequently into the scrotum. This is the natural condition in man; but occasionally it happens that in time the process is, from some cause or another, impeded; and that the testicles either never leave the loins at all, or are stopped at the abdominal rings. Such an impediment is an instance of "arrested development," to use the scientific expression. The full and complete development of the organ, in as far at least as regards its normal situation, has not taken place at the usual time; and the child therefore at and after birth retains a fœtal peculiarity. But this very peculiarity in man is the normal condition in most of the lower animals; and hence we are fairly entitled to adduce the non-descent of the testicles in the human subject as an example of the position which we have laid down—that many cogenital malformations in the child are, as it were, types of the natural condition of the affected parts in some of the lower animals.

In a former part of this article, we have alluded to the "*centripetal*" mode of formation of the uterus, and we then endeavoured to account for the occurrence of the anomaly of a double uterus, by attributing it to an arrested development of the organ, anterior to the coalescence and fusion of its two lateral halves. Whether this interpretation be correct or not, it is especially worthy of notice that this very anomaly affords another very striking example of the analogy which may be traced between the congenital malformations of the human subject, and the normal development of other animals lower in the scale of creation. In numerous tribes of animals the uterus is always bilobed, or bicorned.

The progressive phases in the development, as well as many of the congenital malformations, of the urinary bladder, will also be found to have their types of resemblance in the normal state of the organ in the lower animals. The bladder is in some cases entirely wanting in the human subject, and the ureters have been found to terminate either in the urethra, or rectum, or vagina. Now the comparative anatomist knows that this viscus is absent, not only in all invertebrated animals, but also in most fishes, in many of the amphibia, and in all birds; and if we consult the embryological student, he will tell us that the younger the human fœtus is, the more does its bladder resemble, in shape and situation, the state of the organ in the lower quadrupeds. During the earlier months of intra-uterine life, the bladder has an elongated shape, its fundus being pointed and approaching the umbilicus in the direction of the urachus—which, it is well known, is pervious at this period, and which sometimes continues patent even after birth. Now this description is most correctly applicable to the normal state of the bladder in many of the lower animals.

From the bladder we may pass on to the kidneys; and it will be found that the embryological and comparative anatomy of these viscera also afford a beautiful illustration of the theory, which we are attempting to enforce. In the early fœtus, and in numerous examples of monstrous infants, the kidneys exhibit a multi-lobular appearance—a condition in every respect similar to the normal condition of the organ in the cetacea, &c.

Several of the congenital malformations of the face and mouth are, as already explained, altogether owing to the persistence of an embryotic state of the parts. Now if these malformations be compared with the results of comparative anatomy, it will be found that they are most accurately represented by the structure of the same organs in certain orders of lower animals. The median fissure of the upper lip—one of the most frequent of all congenital defects—is the natural condition of the part, not only in the very young fœtus, but also in all fishes, and in numerous tribes of mammals, besides the family of the hares—which have given a name to the malformation. The fissure of the palate also is present in very many of the lower animals.

The instances now alluded to are so very striking, and withal so generally known, that it is unnecessary to do more, than merely to mention them. There is however an occasional malformation of the bones of the upper jaw, which deserves a more special notice, as its real nature has not been well explained in almost any British work, and as it furnishes another beautiful example of the strict analogy of such defects with the normal state of structure in the lower animals.

We need not remind the student of comparative anatomy, that the most striking difference between the bones of the face in man and those in the lower animals consists in the latter having two bones situated between the anterior parts of the superior maxillæ. These are denominated the 'ossa intermaxillaria.' No such bones are discoverable in the healthy human subject; but in certain cases of double hare-lip a formation, very analogous or rather altogether similar to that of intermaxillary bones, has been found. By a deep fissure through the bone on each side, corresponding in situation to the two lateral clefts of the upper lip, a central or mesial portion is detached from the two lateral portions of the maxillæ; and this central portion is generally found to contain the sockets for the incisive teeth—just in the same manner as these teeth in animals are known to be developed in the genuine intermaxillary bones.

The occurrence of this malformation in the human fœtus is at once satisfactorily explained, if we appeal to the discoveries of embryology. It is to Goethe, the celebrated poet of Germany, that we owe our knowledge of the interesting fact, that in the very young fœtus there are distinct rudiments of intermaxillary bones. The discovery was announced in a memoir, published so far back as the year 1786, and entitled "Dem Menschen wie den Thieren ist ein Zwischenknochen der obern Kinnlade Zuzuschreiben." It at once explains the occurrence of the malformation which we have described above, and it furnishes at the same time a most striking example of the analogy, in point of conformation, of the human fœtus with the other members of the mammiferous class in their perfect state.

If our time and space permitted, we might describe several other occasional malformations of the fœtus, which are strictly analogous to the natural state of the parts in some of the lower animals. For example the tongue is cleft along more or less of its extent in certain cases of monstrosity: in the Phocæ, in serpents, &c. the tongue is always cleft. The penis also is occasionally bifid: this is the normal state of the organ in the Didelpha, Monotremata, and also in the Ophidia. In many of the marsupial tribe of animals, the vagina, as well as the uterus, is divided by a longitudinal septum—a conformation which has been found, in a few instances, in the human subject.

We have not yet spoken of the Nervous system, having purposely reserved the consideration of its development and peculiarities to this part of our enquiry; as we wished to treat of it by itself, in consequence not only of the great importance of the subject, but also of the extreme accuracy with which the brain and spinal marrow have been investigated in the fœtus, as well as in the different classes of animals, by some of the most distinguished anatomists of the present day. We shall now endeavour to give a short abstract of their researches.

No subject of philosophical anatomy is more interesting and instructive than the study of the nervous system in the different classes of animals, and in the embryos of the higher animals during the different periods of their uterine existence. This system, like all others of the body, attains its highest degree of development in the mammals; and among these, more especially in some of the cetacea, in the ape tribes, and in man.

It is however only after passing through a multitude of progressive changes—the types of which are to be found in some of the lower animals—regularly consecutive upon each other, and becoming gradually more and more complicated, that this *maximum* of development is attained.

"The embryo," says M. Serres, "reproduces in miniature, what we find on a magnified scale in some of the inferior animals. The primitive forms of the spinal marrow and of the encephalon are identically the same in all the classes, however much the individuals may vary in point of shape or outward appearance. Thus the embryo of the reptile exhibits at a certain period of its evolution a strictly icthyomorphous nervous system; the embryo of the bird reproduces the type of the reptile's brain, and the embryo of the mammal exhibits successively the encephalic and spinal forms of these three inferior classes."

When aware of these very curious facts, we are at once provided with a solution to many of the congenital anomalies of the brain and spinal marrow, which were utterly puzzling and inexplicable a few years ago. If the human embryo passes through all its stages of evolution without suffering any accident or obstruction, the traces of these primitive and temporary stages are effaced in the perfect fœtus;—but, on the other hand, if the process of development has from any cause been stopped or arrested, the part of the body, which suffered this arrest, remains in the condition in which it was at the supposed period; and then at birth it will be found to exhibit the form and arrangement, which appertains to some animal inferior to it in the scale of organization.

It will be interesting to devote a few pages to a brief description of the progressive evolution of the Cerebro-spinal axis in the different classes of animals; for the reader will thus see what an intimate resemblance may be traced between its conformation at different periods of fœtal life in man, and its permanent forms in some of the lower animals.

We may premise by stating that the Nervous system is—like all other parts of the body, according to M. Serres and many other able anatomists —formed from the circumference to the centre; and therefore that it affords an argument of the truth of the centripetal or excentric theory. The nerves of the trunk, the lateral nerves of the head, and then the nerves of sense are formed before the spinal marrow or the encephalon; and the two lateral parts of the cerebro-spinal axis will be found to be developed from without inwardly, or in other words from the periphery to the centre.

After this prefatory remark, we now proceed to examine the structure of the nervous system in the various classes of animals, beginning with those most simply organized, and gradually advancing up to the highest of the mammifera.

The lowest class in the animal scale has of late years been denominated *Acrita*,* and includes those zoophytic beings, which are grouped under the orders, polygastrica, infusoria, porifera or sponges, polypifera, and acalephæ or sea-nettles. They are characterised as "gelatinous polymorphous animals, without distinct nervous fibre, or visceral cavities. Alimentary canal excavated in the parenchyma of the body, generally without an anus. Generation in most fissiparous or gemmiparous; in some oviparous." Their nervous system, if it does exist at all, is molecular and consists of globules diffused through the cellular tissue of their homogeneous bodies. Some authors indeed have asserted that some of the genera, such as the Actinia, and Beroe, possess traces of an aggregrated form of a nervous organization, as a distinct system; but this statement has not been confirmed by other anatomists; and we may very safely assert that for the most part all the different systems, in the bodies of these most simple animals, are, as it were, blended together into one uniform, homogeneous, granular parenchyma. Professor Carus has denominated the class Acrita, by the name of Protozoa or Oozoa; as being placed on the first step of animal organization, and analogous to the ova or germs of the higher classes; and certainly, although objections may be taken to the propriety of the name, the philosophical

---

* This term is derived from a Greek word, signifying *indistinct*, or, *not to be distinguished*.

anatomist cannot fail to be struck with the wonderful similitudes that may be traced between them.

We shall avail ourselves of Mr. Anderson's very able and ingenious work, to illustrate this most curious theme. After describing the imperfect traces of a nervous system in the lowest tribe of zoophytes, he proceeds to remark:—

"Let us now turn to the consideration of the human ovum: that we know to consist (when first detected) of a thin membrane inclosing a quantity of transparent limpid fluid; it is of that form which has been presumed to be the primary one of all organic matter—the spherical. In a little time the embryo is visible, as a speck, a globule; its texture is soft and homogeneous; no organs or systems are as yet discoverable. Dr. Blundell has in his museum a preparation of the human ovum, consisting of a delicate membranous cyst, in which is seen a little spot, not so big as a mustard-seed—the embryo. And again: Sir E. Home, in describing the appearance of a very early human ovum, says, 'that it consisted of two membranes, which contained (besides a slimy fluid) two globules—the rudiments, probably, of the heart and brain.' Even during the first month of human embryotic life, the minute researches of Tiedemann record 'that no organ is yet perceptible,—that the cephalic and cervical swellings are altogether transparent, and contain only a limpid fluid.' The embryo state of the lower animals presents similar phenomena. I have had the opportunity of examining a very early embryo of the squirrel, only a line in length, and scarcely half a line in breadth, which was of an elongated spherical form, of an apparently homogeneous texture. The embryo of the common fowl, on the first and second days of incubation, I have found also to consist of a spherical globule or vesicle. Again: the ovum of the frog has a spherical form, and is of a homogeneous pulpy texture." 6.

Ascending in the scale of animal organization from the class Acrita, we come to the class Radiata, which includes the asteriæ or sea-stars, the echini or sea-eggs, the holothuriæ, &c. In them nervous filaments—formed by a longitudinally arranged series of nervous globules—are for the first time discoverable. These filaments are so disposed as to form a primary nervous ring round the mouth of the animal, and from this ring proceed minute threads to the different divisions or rays of the body. Such is therefore the arrangement of the nervous system in its simplest form.

In the Mollusca, the third class of the animal kingdom, (if we assume the nervous system as the basis of our classification) we find that, in most of them, the apparatus of nerves has acquired a much higher degree of development. Besides the primary nervous ring, we find two ganglia, one situated above, and the other below the œsophagus. From the former, the supra-œsophageal, are given off the nerves to the eyes, the mouth, the tentacula and the organs of generation; and from the latter—the sub-œsophageal—arise the nerves which supply the bulk of the body, its external tunics, most of the viscera, &c. The supra-œsophageal ganglia is almost always considerably larger than the sub-œsophageal one, and is the earliest rudiment of that most important part of the nervous system, the *brain*. Hence it is sometimes called the *cerebral* ganglion. Mr. Anderson's description is, as follows:—

"The supra-œsophageal or cerebral ganglion is the first appearance of a medullary mass corresponding to a brain; and from it arise the optic nerves, already of large size. It must therefore be considered as analogous to the tub.

quadrigemina of the higher animals, and is indeed the first lineament of that cerebral mass which we shall trace through successive complications to the perfect brain of the human species. In fact, in the Cephalopoda, the highest of the Mollusca, we find this cerebral ganglion divided into anterior and posterior lobes, similar to the tub. quadrigemina of the Mammalia; it is of much greater relative size than the lateral or inferior ganglia developed on the primary nervous ring, which, indeed, have here almost entirely disappeared, and have given place to simple largely-developed commissures. Here, then, we have arrived at the highest degree of complication and development of the simple primary nervous ring; and in the next group, the Articulata, we shall find this same highly-developed ring multiplied several times." 9.

The fourth class of animals, in the ascending scale, is that of the Articulata, among which we include the Annelida, such as the common earthworm, leech, &c. the Insecta, the Arachnida, the Crustacea, the Cirrhopoda, or the tribe of barnacles, &c. It is characterised by the circumstance of the body of the animal being divided into a certain definite number of segments, which correspond to certain divisions of the nervous system, and each of which may be considered as a repetition of the rest. Of these segments the most anterior acquires the greatest development and is called the head.

As a general remark, we may state that, in every segment, there exists a nervous ganglion, and these successive ganglia are connected with each other in the median line of the body by a double longitudinal filament—thus exhibiting the earliest rudimentary example of a spinal cord. If we take for example the Aphrodita, vulgarly known by the name of the sea-mouse, we find the following arrangement of the nervous system.

" It occupies the middle line of the ventral aspect of the body, and consists of a double series of minute ganglia of medullary matter, more or less intimately united or even blended together, and equal in number to the number of rings, of which the body is composed. Hence the appellation of Annelida to the order of animals, comprehending the worms, leeches, Aphroditæ, &c.

The ganglia give origin to lateral branches, and are connected together by two cords of communication, sometimes separate, sometimes united into a single trunk, so as to constitute a longitudinal chain extended through the entire length of the body. The first of these ganglia is lodged in the head, or at least at the anterior extremity of the animal, in front of, or above the digestive tube (the supra-œsophageal or cerebral ganglion); the rest are placed below that canal; whence it results that the two nervous cords, which form the media of communication between the cephalic ganglion and the first of the sub-œsophageal series pass along the sides of the œsophagus, and form around that canal a species of collar or ring;—a character which is common to all the articulate animals."

This description is equally applicable to the worm, leech, &c. Such therefore is the arrangement of the nervous system in the lower tribes or orders of the Articulate animals. In the higher orders, such for example as the Crustacea, Insecta, and Arachnida, the organization acquires a greater degree of evolution; the cerebral ganglion becoming more and more developed, and the other ganglia being more determinate as to number and more concentrated as to situation. This gradually-increasing complexity of structure may be traced from the Crustacea to the Myriopoda or centipedes, from these to the Arachnida or true spiders, and thence to the Insecta, the last and highest order of the Articulata, " in which," says Mr. Anderson, " we find the nervous system very highly organized, leading us by strict analogies to the Vertebrata. I have found it to consist, in almost every order, of a ganglionic nervous cord, running along the abdominal surface, as in the preceding classes, and of a similar supra-œsophageal nervous mass called by Cuvier the brain, from which are given off eight pairs of nerves and two single ones. This nervous cord consists of a varied number of ganglia giving off lateral nervous filaments, and connected to each other by longitudinal sensitive columns. A motor tract has also been recently described by Mr. Newport, passing along the dorsal surface of these columns, and giving off lateral nervous branches. Minute anatomy has also unfolded to us what may be considered as the analogue of the respiratory system of nerves, and a par vagum."

In no part of the nervous system is the increase of development so conspicuous, and for the illustration of our present enquiry so important, as in the supra-œsophageal or cerebral ganglion. We have already stated that this is the analogue, or rudimentary representative of the brain in the higher animals. We have shewn that in the lower tribes of the class Articulata it is a simple ganglion, similar to and only somewhat larger than the other ganglia of the body; but when we arrive at the proper insects and examine it in them, we find that it exhibits a positive division into lateral lobes or hemispheres; that it preponderates greatly in size over the other ganglions; and that the sensorial nerves, which arise from it, begin to present distinct ganglionic enlargements.

Before proceeding to examine the development of the cerebro-spinal axis in the Vertebrata, let us here pause a little, and compare the preceding details with the several transitory conditions or phases of this apparatus in the successive periods of evolution in the higher animals, during their embryotic existence, and we shall discover some very remarkable analogies which have been revealed to us by the labours of philosophical anatomy. The following extract is a long, but a valuable one; and we shall not therefore hesitate to lay it before our readers.

"The anatomical details that have hitherto been made respecting the nervous system are increased in interest when we turn to the consideration of the early human embryo, where, although no such *positive* similarity of conformation exists, as I shall hereafter demonstrate, between the advanced fœtus and the Vertebrata, yet strong analogies in structure and development may be pointed out. I have already noticed the early human ovum before any embryo was apparent, and have pointed out the analogy existing between it and the seed of plants, the lowest Infusoria, the monads, &c. I have also spoken of the early embryo itself as a minute spherical homogeneous mass, where the nervous system was supposed to be molecular, as in the Acrita, the lowest Tunicata, and the lowest Entozoa. As the embryo increases in development, nervous filaments are discovered, formed (as we conceived in the Radiata) by a longitudinally-arranged series of globules, these nerves, doubtless sympathetic ones, are then formed before the spinal cord and brain, and previous also to their communication with those two organs—which phenomenon Tiedemann considers best explained by the general law of organization laid down by M. Serres, viz.—that the development of the nervous system proceeds from the circumference to the centre. Now, on reflecting on the state of the nervous system of the animals already described, we find that filaments exist in many of them, without any approach to the character of a spinal cord—a mere ganglionic sympathetic system. Arrived, however, at the Articulata, a longitudinal arrangement of closely-approximated ganglia is observed, forming a double nervous cord, extended along the ventral surface of the animal, at the upper extremity of which, on the dorsal aspect, a cerebral ganglion is developed. This is highly interesting, from the similar rudimentary condition of similar parts in the human embryo, the brain and spinal marrow, the first visible rudiments of which (according to Tiedemann) appear between the fifth and sixth week, in the form of a whitish vesicular fluid, contained in a membranous canal or tube, in the trunk leading to or forming a cavity or pouch in the head. At the seventh week the spinal cord is large and thick, the rudiments of the two lateral columns of which are manifested in the form of a simple pulpy streak—a nervous streak greatly similar to what I have described in the Ascaris, amongst the Entozoa; at the upper extremity it forms, or is contiguous with, a mass of medullary matter, the most developed portion of which may be considered as analagous to the cerebral ganglion of the articulata and gasteropodous Mollusca, which itself represents the optic lobes of the inferior Vertebrata, the tub. quadrigemina of the Mammalia." 11.

"With regard to any analogy between this state of development of the nervous system in the Articulata and in the human embryo, little more can be observed than that the structure of the ganglionic abdominal cord in the Insecta bears a strong analogy to that of the spinal cord in the Vertebrata generally, and in man individually; that the high development of the cerebral ganglion, its division into lobes, and the ganglionic enlargements of the sensorial nerves, may be considered as analogous to the early and great development of the tub. quadrigemina in the human embryo, and the early appearance of the optic nerves." 16.

We are now prepared to pass to the examination of the nervous system in Vertebrate animals, that great class or division of the zoological scale which includes fishes, reptiles, birds, and mammals, and of which man is the most exalted member in point of development and organization. We have already pointed out certain analogies which may be traced between the nervous system of Invertebrate animals, and the early or primitive condition of this system in the human embryo. But these analogies, although full of marvellous interest and most deserving of attention by the philosophic physiologist, are at best but vague and glimmering. Not so however with those, which may be easily shewn to exist between these embryotic conditions, and the normal and perfect conditions of the brain and spinal cord in the successive classes of Vertebrate animals, from fishes up to the quadrumana among the mammals. They are not merely *analogies*, but strict and most indubitable *resemblances*. They are almost exact counterparts, the one of the other. It will appear, for example, that, at a certain period of fœtal existence, the human brain exhibits the appearance of this organ in fishes; and that, as it advances in development, it repeats, so to speak, the normal condition of the same part in reptiles, in birds, and in the lower mammals, until at length it reaches its perfect formation, such as we see it in the full-formed child.

The mere mention of so curious and instructive a subject as this cannot surely fail to excite the attention of all who feel an interest—and who is there of enlightened education who does not?—in searching out the wonders of Creative Power.

How strange, how marvellously beautiful are the works of Nature! The more we study them, the more we are lost in admiration at the countless variety of her productions, and at the simple and harmonious laws which guide and direct all! Nowhere do we find more vivid proofs of the 'multiplicity of effects from unity of cause' than in the study of the development of the vegetable and animal kingdoms. We trust that the short and imperfect exposition of the subject, which it is our aim to give in the present article, may serve to diffuse a fondness for philosophical anatomy, a science hitherto so much neglected in this country. With the exception of a very few recent labourers—and of these perhaps the most meritorious are Professor Grant, Mr. Owen, Mr. Newport, and we shall add for the sake of encouragement Mr. Anderson—our knowledge of this delightful study is altogether drawn from the labours of the German and French physiologists. To Tiedemann and to Serres, we are most largely indebted; and it is from their works that we derive most of the following details. Before describing the structure of the cerebro-spinal axis in the Vertebrate animals, it is proper to premise that, in all the classes, the encephalic portion consists of three divisions or segments—an anterior portion or cerebrum, a posterior portion or cerebellum, and a median portion or the mass of the tubercula quadrigemina. Thus, says Mr. Anderson, the number of ganglia forming the brain, the most highly-organized part of the nervous system, is definite and invariable; while the number of ganglia forming the spinal cord, the least organized part, is indefinite and variable.

In Fishes, the lowest order of the class, the following may be taken as a general description of the cerebro-spinal axis.

"The spinal cord is remarkable for its great relative size in this class of animals; it is continued (with but very few exceptions) the whole length of the vertebral column, even into the caudal vertebræ, and it has on its anterior and posterior aspects a longitudinal fissure, the latter being the deepest; internally it is hollowed out by a canal, which traverses it in its whole extent, and which, at the upper part, immediately posterior to or underneath the cerebellum, forms a considerable dilatation or enlargement—the fourth ventricle. In a river lamprey (*Petromyzon fluvialis*), weighing 570 grains, I found the brain to weigh only four-tenths of a grain, while the spinal cord weighed three grains, the proportion being as 100 : 750. We thus observe how much the latter preponderates in size, being seven and a half times heavier than the brain." 17.

The appearance of the brain itself varies a good deal in the different tribes of fishes. In all, it is of very small dimensions, in reference not only to the size of the animal, but also to that of the spinal marrow.

"Its very small size is at once evident by comparing its weight with that of the whole body of the animal; thus I found in a chub weighing 842 scruples, the brain weighing only one scruple, the proportions being as 100 : 84,200; in a carp, weighing 11,280 grains, the brain weighing only fourteen grains, the proportions being as 100 : 80,600; in a roach, weighing 5030 grains, the brain weighing only nine grains and a half, the proportions being as 100 : 52,900; and, as I before observed, in a lamprey weighing 570 grains, the brain weighing only four-tenths of a grain, the proportions being as 100 : 142,500. We thus observe how little relative size the brain bears to the rest of the body, and consequently how small is as yet the encephalic mass. On taking a general review of the conformation of the cerebral masses forming the brain of fishes, we find it to consist of a suite of ganglia arranged behind each other—two pairs and a single one:—1st, there are two ganglia or lobes, situated the most anteriorly, the olfactory lobes; immediately behind which are two others, generally of larger size, the optic lobes; and behind these, again, is a single ganglion or lobe, situated in the median line, the cerebellum." 18.

The olfactory or anterior ganglia are considered, by the best anatomists, as the analogue or representative of the cerebral hemispheres in the higher animals; and the optic or middle ganglia as that of the tubercula quadrigemina. The latter bodies are generally of large size in fishes, and contain internal tubercles and cavities, which communicate with the fourth ventricle. The cerebellum is imperfectly developed.

Let us now compare with the structure described, the state of the spinal cord and brain in the human fœtus at an early period of its intra-uterine existence.

In the *third* month the spinal cord is a hollow cylinder of nervous matter, having a longitudinal groove on its anterior and posterior aspects, and containing a central canal, which traverses its whole extent, and, as in fishes, dilates at the upper part to form the fourth ventricle.*

Its volume, in proportion to that of the brain, is much more considerable at this period, than it is afterwards; and moreover it extends to the very extremity of the vertebral canal, in the sacrum and coccyx, and exhibits no trace of cauda equina. The same arrangement is found in fishes; the medulla in them being prolonged into the extreme point of the caudal extremity of the animal, and terminating in a point.† The superior portion of the cord, the medulla oblongata, is large and broad; the pyramidal fasciculi are seen continuous with the crura cerebri, as in fishes; and the restiform bodies, after separating to form the fourth ventricle, pass into the cerebellum.

So much for the spinal marrow. Now with respect to the encephalic portion of the nervous centre. Mr. Anderson says:—

"This description of the first cerebral mass in the fishes will be accurately represented in the human embryo at the middle of the third month, at which time, according to Tiedemann, the cerebral hemispheres, or *first cerebral mass*, or olfactory tubercles, consist of two membranous vesicles covering the corpora striata and the optic chambers partially. The tub. quadrigemina and the cerebellum are quite exposed; their surfaces are quite smooth, and free from convolutions." 19.

"Turning to the consideration of the *second cerebral mass*, or optic lobes, or tub. quadrigemina, in the human fœtus at the age before spoken of, (the third month) we find that they consist (according to Tiedemann) of two proportionally large distinctly-separated masses, uncovered by the hemispheres, containing a large cavity, and giving partly origin to the optic nerves." 20.

And lastly, of the cerebellum, he adds "that the permanent form of

---

* This central canal of the spinal marrow has been occasionally met with in the human adult by Morgagni, Malpighi, Haller and others. Such an anomaly must be considered as the result of an arrested development, as no such canal exists in the perfect state.

† M. Serres and other embryologists assure us that, at this early period of intra-uterine existence, there is actually a caudal prolongation of the coccygeal vertebræ in the human fœtus, and that this *tail* is gradually absorbed, just in the same manner as the tail of the tadpole is removed by absorption during the transformation into the frog. In the tadpole, the spinal cord is found to be prolonged through the coccygeal vertebræ; but, as the metamorphosis proceeds, and as the tail becomes absorbed and the extremities are developed, a gradual retraction of the cord takes place. M. Serres says that he has observed similar changes in the human fœtus.

this part in many tribes of fishes is accurately represented in the human fœtus at the *third* month."

Advancing in our comparative investigation of the development of the nervous system, we now proceed to state its condition in the next class of vertebrate animals, the Amphibia and Reptilia. The brain and spinal marrow in these animals bear a great similarity in structure and development to those of fishes. As in them, the spinal cord still preponderates greatly in volume over the brain, extends down (except in the adult frog) the whole length of the caudal vertebræ, contains a central canal which communicates with the fourth ventricle, and is deeply fissured by a longitudinal groove on its anterior and posterior surfaces.

On examining the brain, we find, as before, three principal parts to occupy our attention—the olfactory tubercles, the optic lobes, and posteriorly the cerebellum. With respect to the first or olfactory tubercles, they now become obviously the cerebral hemispheres; they are of an increased proportional size, commencing to cover the corpora quadrigemina; and they are found to contain a cavity. The optic lobes are of small size and are more solid than the same parts in fishes; their internal cavity being smaller and their general conformation approaching more to the form and character of the tubercula quadrigemina of the mammalia.

The condition of the cerebro-spinal axis in the human fœtus, which may be considered as analogous to and represented by the permanent condition in the class now described, is exhibited during the *fourth* and beginning of the *fifth* months of embryotic life. Tiedemann assures us that, up to the sixth month, the spinal cord contains a large central canal, similar to that found in reptiles. We also observe that it gradually diminishes in relative size—a consequence of the increased development of the encephalon.

It is unnecessary to particularize the analogies, which are to be traced between the encephalon of the early fœtus and that of reptiles, after what we have stated respecting the structure in fishes.

We proceed therefore, up the scale of organization, to birds.

In them, the spinal cord although of less relative size and of less extent than in fishes and reptiles, is still, as in them, traversed by a distinct anterior and posterior longitudinal fissure, and it still contains a central canal.* The proportional volume of the cord and the encephalon is now so much altered, that the latter is found to preponderate over the former—a most convincing proof of exalted development in the nervous system.

Very analogous is the state of the cord in the human fœtus in the *fifth* month. It cannot be traced lower than the sacrum, terminating there by a slender filament. As already observed, it still also contains a central canal.

With respect to the brain in birds, it is found to exhibit a similar number of parts—the anterior, olfactory, or cerebral masses, the optic lobes, and the cerebellum—as in reptiles; but the parts are more highly developed, and are no longer arranged in a longitudinal series as formerly, but are situated more above, or on the top of, each other.

---

* The spinal marrow in birds does not descend lower than the sacrum; that portion of it, which passes through the coccygeal vertebræ, being considered by many anatomists as only a large terminal filament.

The anterior masses are now distinctly recognised as constituting the proper cerebral hemispheres; they are of greater relative size than any other parts of the brain; they are united to each other by a commissure, the anterior commissure, and above this by another commissure, which Meckel regards as the rudiment of the corpus callosum; they contain cavities, which are true lateral ventricles, and in them we discover a tubercle or enlargement, corresponding to the corpus striatum in mammals.

The optic lobes or second cerebral mass are of small size, and are more separated from each other, than in the preceding classes. Each of them contains a small cavity, which communicates with the third ventricle. The cerebellum has now attained a great degree of development, and bears a striking analogy to this part in the higher animals. It consists of a more or less rounded median lobe, with very small lateral appendages; its external surface is marked by transverse sulci; and, on being divided, it exhibits traces of the arbor vitæ.

The following extracts from Mr. Anderson will exhibit the analogy of the structures, now briefly noticed, with the state of the fœtal brain in the fifth and sixth months.

"At that time, according to Tiedemann, the cerebral hemispheres, or *first cerebral mass*, are smooth and free from convolutions, as I have myself observed in two fœtuses of that age; they have a more convex oval form, and are developed backwards so as to cover the superior surface of the tub. quad., leaving the posterior surface exposed, as I have just described in the birds. The anterior commissure exists, as also the corpus callosum; but this latter is very small and narrow, as I have myself observed."

"With regard to the tub. quad., or *second cerebral mass*, in the human fœtus at the age before spoken of (the fifth month), we find they are smooth and more convex, still hollow, though the cavity is somewhat diminished, and presenting no traces of the four eminences which are subsequently to be developed. The optic lobes in birds, their analogues, have also been described as two smooth, convex, hollow masses, but in which the internal cavity was less than in the preceding classes. Their situation with regard to the cerebral hemispheres appears different from the human fœtus, but the quantity of surface uncovered by them certainly bears a great analogy of development." 25.

"In the human fœtus at the fifth month the cerebellum is much increased in size, and for the first time, according to Tiedmann, 'presents evident traces of the division into a central part and two lateral hemispheres;' for the first time, also, 'transverse grooves, four in number, are perceptible, dividing the organ into five lobes, which represent in a perpendicular section exactly five stems: the branches or leaflets do not yet exist, consequently the appearance of the arbor vitæ is but faint.'" 26.

We are tempted to extract also the following observations, containing a resumé of the whole.

"On reviewing these statements of the nervous system in the birds, and in the human fœtus at the fifth month, we observe that the brain and spinal marrow are no longer situated on the same horizontal plane, and that the preponderance is now in favour of the brain: its weight, too, when compared with the body, is greater; and the ganglia composing it are more above, and less behind each other. The primary cerebral mass has now acquired so high a degree of development as to surpass the others in size; no convolutions are, however, yet apparent on its surface; no large commissure yet exists to unite them. The optic lobes, or median cerebral mass, are small, separated from each other, and their cavities have decreased. The cerebellum, or third cerebral mass, is large; traces of lateral lobes are evident, and external striæ are perceptible." 26.

We now arrive at the last and highest class of the animal kingdom, the mammalia, to which man belongs, and in which the nervous as well as all the other systems of the body acquire their most complicated and perfect development. The transition from the preceding class in point of organization is very gradual and not marked by any abrupt or very distinctive changes. The spinal cord for example, although of less relative size than in any of the preceding classes, exhibits the same conformation; it is still traversed by the anterior and posterior longitudinal fissures, but these fissures are less deep; and it still exhibits a central canal in all, except in man. It does not descend so low down in the vertebral canal as in birds, but terminates usually about the lower lumbar vertebræ in a true cauda equina, or leash of nervous filaments.

It is in the structure of the encephalon however that we discover the most conspicuous proofs of a more exalted development. Its direction with respect to the spinal cord is no longer horizontal or in the same line, as it is in fishes and reptiles, but approaches more or less to a right angle; the first traces of which inflexion were perceptible in birds. Its relative size and bulk are greater than in any of the preceding classes, and the different parts, of which it consists, are more numerous and more complicated. We shall now briefly notice these changes, as they affect the cerebral hemispheres or first cerebral mass, the optic lobes,* or second cerebral mass, and the cerebellum.

1. The cerebral hemispheres, or anterior mass, have now acquired a high degree of development. They are of a large size, compared with the rest of the encephalon; but this relative size varies much in different tribes. In some, these hemispheres very much resemble the same parts in birds; being small, and presenting no traces of convolution on their surface, and leaving the tubercula quadrigemina quite exposed. This is the case in the dolphin, in the bat, in the rabbit, rat, mouse, &c. In the pig, horse, ass, sheep, and deer, they are proportionally larger, extending backwards so as to cover the tubercula; and moreover the surfaces are convoluted. In the monkey tribe, they are still larger, more rounded and elevated, broader in the middle, and extend further backwards, so as to cover the cerebellum; the convolutions also are more numerous than in the other classes; the fissure of Silvius is deeper; and we first discover the existence of posterior lobes, as yet but of small size, and unconvoluted. In the ourang-outang they are altogether larger, and approach more nearly the form and character of the human brain, covering the cerebellum entirely, and convoluted on their posterior lobes.

The corpus callosum, or great commissure of the cerebral hemispheres—which makes its first appearance in mammiferous animals—varies much in size in the different tribes of this class.

---

* The reader should well remember that the parts denominated the optic lobes are not the thalami optici—a mistake which the resemblance of the names is apt to produce—but rather the corpora quadrigemina, from which the nerves of vision arise.

It is very short in the bat, rabbit, rat, mouse, &c., longer and broader in the pig, ass, sheep, &c., and still more developed in the quadrumana, attaining its maximum of development in man.

With respect to the lateral ventricles of the brain, they are proportionally larger in the inferior groups of mammals; becoming more contracted and smaller in relation to the size of the hemispheres, in those animals which approach the human species.

If our space permitted, we should allude to the gradually increasing development of other parts, such as the cornua of the ventricles, the corpora striata, fornix, thalami optici, &c. But the investigation of these minutiæ we must leave to the curiosity of the reader. We must not, however, omit to mention a peculiarity of the olfactory nerves in most mammiferous animals, as it furnishes another very beautiful instance of the exact similitude between the permanent development in the lower animals, and one of the transitory and successive phases of development in the human fœtus.

These nerves are hollow and their cavities communicate with the lateral ventricles. Now this very state of parts exists in the human fœtus up to the seventh month of gestation.

2. The optic lobes, or second cerebral mass, (or, as they are now to be called, the tubercula quadrigemina) consist in all mammals of an anterior and posterior pair of ganglia, which vary much in size and in position. In the lower mammals, the anterior pair are the largest, and are bulky, compared with the cerebral hemispheres; in the horse, sheep, deer, &c. the anterior are still the largest, but they are of less proportional size to that of the brain; in the monkey the two pairs are nearly of equal size, and exhibit less relative volume—thus approaching very much to the characters of the tubercula quadrigemina in the human adult brain.

3. The cerebellum, or third cerebral mass, exhibits great differences in the size of the mass itself, and of its different parts, according as the animal is high or low in the development of its encephalon. It is largest, compared with the brain, in the bat, rabbit, rat, &c.; somewhat proportionally smaller in the sheep, deer, horse, &c.; and very considerably smaller in the monkey. The division of its mass into lateral and median lobes is better marked, and the convolutions or the striæ on its surface are more numerous and more distinct, as we rise in the scale of organization. Hence these characters are more conspicuous in the quadrumana than in any of the lower tribes and approach, in their development, very nearly to the appearance of the human cerebellum.

The rapid review, which we have thus taken of the gradually-increasing complexity in the development of the cerebro-spinal axis in the different orders of the mammiferous class, from the rodentia up to man himself, will enable the reader to trace the very striking analogies which may be traced between these various forms and the successive phases which the human brain undergoes during fœtal life, until at length it attains its mature development.

With respect to the spinal marrow, there is little more to be added to what we have already stated. We have shewn that, in the young embryo it descended as low in the vertebral canal as the extremity of the sacrum, and that it contained a central longitudinal canal.

As the fœtus advances, the cord becomes gradually more and more retracted upwards, until at length, in the ninth month, it does not descend lower than the third or fourth lumbar vertebræ. As this change—a change altogether analogous to that, which the spinal cord of the tadpole undergoes during its metamorphosis to the state of a frog—is going on, the cauda equina becomes more and more distinct, and the central longitudinal canal becomes narrower and narrower, until at length it is obliterated during the eighth month.

No less striking are the successive changes which the human cerebrum and cerebellum pass through, in their progress to perfect evolution.

We have already seen that the state of the fœtal encephalon at the *fifth* month is very analogous, in many respects, to that of the organ in birds. The cerebral hemispheres are comparatively small, unconvoluted, and not covering the tubercula quadrigemina. The fissure of Silvius is not deep or distinctly marked. This description is strictly applicable to the brain of the bat, rabbit, and most of the Rodentia. In the *sixth* month, these hemispheres have enlarged so much that they cover the tubercula, and their internal surface begins to exhibit grooves, the rudiments of the convolutions. These marks of increased development are met with also in the pig, horse, sheep and cat. In the *seventh* month, the cerebral hemispheres are so much increased in size, that they extend even a little beyond the cerebellum; their surface moreover exhibits depressions here and there; and the fissures of Silvius have become considerably deeper. At the *eighth* month, the anterior, middle, and posterior lobes are well defined; the grooves, depressions, and convolutions have become deeper and more numerous, excepting on the posterior lobes—and this is found to be the case in the brain of the quadrumana.

The gradual development of the corpus callosum in the fœtal brain furnishes other proofs of similar and most interesting analogies. In the *fifth* month, it is only just apparent, such as we find in the brain of birds; at *six* months, it is very short and narrow, as in the bat, and in the lower Rodentia; at *seven* months, it is larger, covering the optic chambers and third ventricle, as in the Pachydermata, Solipeda, Ruminantia and Carnivora; and at *eight* months, it extends to the anterior pair of the tubercula quadrigemina, as in the Quadrumana.

If we next attend to the lateral ventricles, their characters also vary much at different periods of fœtal evolution. In the *fourth* and *fifth* months, they are very large, as in reptiles and birds; and we find that they become proportionally smaller and smaller, as the fœtus advances—thus typifying, if we may use the expression, the condition of these cavities in the different orders of the mammiferous class.

The communication, existing between the anterior cornua and the cavities in the substance of the olfactory nerves up to the seventh month, has been already alluded to.

Let us now direct our attention to the gradual evolution of the second cerebral mass, the Tubercula Quadrigemina.

"The tub. quadrigemina, or *second cerebral mass*," says Mr. Anderson, "in the latter months of the human fœtus pass through a similar grade of development. I have already spoken of their organization up to the fifth month, when they were compared to the same parts in the birds, and may now be compared to the Cheiroptera and Rodentia amongst the Mammalia. At the sixth month, according to Tiedemann, they are quite covered by the cerebral hemispheres, and their internal cavity is diminished; at the seventh month they are divided into two pairs of ganglia, the nates and testes,—the internal cavity is nearly obliterated, and the communication of the fourth ventricle with the third is reduced to a mere canal, constituting the aqueduct of Sylvius, and their structure altogether is the same as at the eighth and ninth months, and as in most of the higher Mammalia." 30.

The development of the cerebellum presents equally striking proofs of the analogies, which we have so frequently spoken of. It is therefore unnecessary to detail them.

We have thus completed our examination, founded on the results of comparative and of embryological anatomy, of the nervous system; and we trust that our readers will experience the same delight, which we have felt ourselves, in studying the operations of living nature, and in being made to know the steps, as it were, by which she works in building up the marvellous machinery of our own bodies. It may be useful to recapitulate briefly the most important conclusions which may be drawn from the preceding descriptions.

" 'These are,' to borrow the language of Mr. Anderson, 'that Nature follows an uniform plan in the creation and evolution both of the brain of the human fœtus and of that of vertebral animals.'

2. That the brain of the human fœtus, at certain stated periods during its passage from a simple to a complicated state, is twice represented in nature; that is, by a permanent form, and by a transient form of the brain of a lower class of animals. Thus, the human fœtal brain at the fourth month, is permanently represented by the reptiles, and transiently represented by the fœtal chick at the sixteenth day, and the fœtal sheep at the eighth week.

3. That the analogies in the structure of the brain in the human fœtus, and in animals, are uniform in their existence, and are applicable to each portion of the cerebral mass, at one stated period of its development in man, and in one particular class of animals. Thus, the analogy in the conformation of the human fœtal brain at the fourth month, and in the reptiles, exists in each portion of the cerebral mass, and not in one particular portion only.

4. That the definite periods at which these analogies are most apparent may be established as under.

We may also reasonably infer, that similar analogies exist, and similar modes of development are pursued, in the evolution and perfection of every other organ or system in the body.

| *Human Fœtal Brain.* | *Brain of Animals.* |
|---|---|
| 3d month.......... | Fishes |
| 4th month .......... | Reptiles |
| 5th month.......... | Birds |
| 6th month.......... | Mammalia (Ruminantia, Carnivora, &c. |
| 7th month.......... | Mammalia (Lower Quadrumana) |
| 8th month.......... | Mammalia (Highest Quadrumana) |
| Ninth month." | |

Here therefore we shall stop in our examination of those analogies, which may be traced between the transitory formations of the fœtus, and the permanent formations in the different tribes of lower animals.

There is still another topic of Embryological anatomy, to which we now wish to direct the reader's attention—we allude to the evolution of the generative organs, and to the explanation, which the study of this affords, of those singular irregularities of structure, to which the term of Hermaphrodism has been applied. The subject is one of very great interest, not only as being illustrative of some of the more remarkable doctrines of philosophical anatomy, but also as involving some questions of practical importance. We have deemed it better to introduce the discussion of it at this than at an earlier part of our paper, as the details, which we are about describe, might have distracted too much the attention of the reader from the general positions, we were endeavouring to establish. It may be useful to premise that the development of the generative organs in both sexes is beautifully accordant with and illustrative of M. Serres' theory—that of the Centripetal or Excentric formation of the body. Some of the parts of this apparatus are, it is well known, single, others are double; but they all agree in being strictly and uniformly laterally symmetrical. The one lateral half is in every respect the counterpart of the other lateral half. The cause of this uniform symmetry in the single, as well as in the double, organs is at once readily explained by bearing in mind, that in the young fœtus the former consist of two lateral halves, developed apart from each other, and ultimately coalescing along the mesial line. Now almost all the structural irregularities of the sexual organs, in other words, almost all the varieties of Hermaphrodism will be found to resemble most accurately the embryotic conditions of these organs, at different periods of intra-uterine existence.

All cases of Hermaphrodism may be conveniently and very appropriately arranged under two great classes—into the "hermaphrodisme sans exces," and the "hermaphrodisme avec exces;" or into Simple and Compound Hermaphrodism.

In the *former*, the actual number of the generative organs is not changed; and the anomalies, which they may exhibit, appertain only to the configuration and structure of the parts. In the *latter* class, the number of these organs is positively increased, either by certain of the male parts being superadded to the female apparatus, or vice versâ. It is of the greatest importance that this two-fold division of Hermaphroditic malformations should be attended to in examining and describing any individual case, as the two classes are separated from each other by well-marked and very natural distintive characters. To Meckel, the distinguished German anatomist, is due the merit of having first accurately pointed out and ably illustrated the classification of Hermaphrodites.

The varieties of simple Hermaphrodism—"H. sans exces"—are very numerous. In some cases the structural irregularities are so inconsiderable, that we have no difficulty in at once discovering the sex of the infant. The *ensemble* of the generative apparatus is decidedly either masculine or feminine; one or two only of the subordinate parts exhibiting perhaps the opposite or inverse sexual condition. In other cases, on the other hand, the confusion is much greater and consequently is more perplexing. This may depend *either*, upon all the external organs being, as it were, half male and half female, an apparatus essentially male having a tendency to assume the characters of the female, or an apparatus essentially female having a similar tendency to assume the characters and type of the male; *or*, upon some parts of the apparatus appertaining strictly to the male sex, while other parts of the same apparatus appear to be as truly appertaining to the female.

M. St. Hilaire has, in accordance with these differences now described, arranged all the cases of Simple Hermaphrodism in four subdivisions—the *masculine*, the *feminine*, the *neutral*, and the *mixed*. In the first, the apparatus, although malformed, is essentially and obviously male; in the second, it is as obviously female; in the third, the parts have assumed characters intermediate, as it were, between those of the male and of the female; and lastly, in the fourth, certain parts seem to be strictly male, while other parts seem to be as strictly female.

The second great division also of Hermaphrodism—that which is characterised by the circumstance of the number of the generative organs being actually increased, and in which there are certain supernumerary parts present, while the rest of the apparatus may be either normal or not—has been conveniently subdivided into several groupes, according to the more prominent characters of the case. Thus, if the *ensemble* of the apparatus is decidedly male, and to this male apparatus one or more of the female organs are superadded, the anomaly has been called "Masculine Compound Hermaphrodism;" if on the other hand, certain male organs are co-existent with an apparatus truly and essentially female, the case is one of "Feminine Compound Hermaphrodism;" and lastly, to the extreme cases, where there are two distinct apparatus, one female and the other male (more or less incompletely developed) in the same body, the term "Bisexual Hermaphrodism," has been applied.

Such is a short notice of the classification of Hermaphroditic malformations. We shall now proceed to examine whether any rational conjecture can be formed as to the mode, in which these strange structural irregularities take place. There is not one subject in the whole range of physiology, which, until of late years, was so impenetrably mysterious and so utterly bewildering to the student of nature, as the etiology of hermaphrodism; and, although much progress has been made by some of the ablest French and German anatomists of the present century in this field of embryological science, and their discoveries and researches have taught us to explain the manner of occurrence of some of the irregularities in the development of the generative apparatus, we are still far, very far from being able to give a philosophical interpretation of the whole subject.

It has been to the successful study of comparative anatomy in the first instance, and subsequently to that of embryogeny in the different classes of animals, that we are mainly indebted for the light which, of late years, has been thrown on this obscure path of enquiry.

The labours of the comparative anatomist have discovered that, in certain tribes of animals, the male and the female organs of generation differ so little from each other in appearance and in formation, that they may be fairly considered not only as analogous, but as almost exactly similar, to each other. This resemblance is in some instances so great that difficulty is experienced in distinguishing the two sexes.

While comparative anatomy teaches us this important fact, the study of the successive developments of the embryo has led to the wonderful discovery, that at a very early period of intra-uterine existence the male and female organs are so strikingly alike, that the sex of the fœtus cannot with certainty be predicated.* It is this anatomical analogy—so beautifully illustrated by the researches of the elder St. Hilaire, of Blainville, Meckel, Serres, and some other continental writers—which is the key to the solution of almost all the irregularities or malformations in the development of the sexual apparatus. For, if each part of the apparatus in one sex is essentially analogous in its elementary composition and evolution to a corresponding part of the apparatus in the other sex, and if their subsequent differences result simply from certain differences (the nature of which, we must confess, is quite beyond our knowledge) in the mode or in the degree of their evolution, nothing is more easy than to conceive the existence of a state of development intermediate between that of the one sex and that of the other. For example, if the clitoris may be regarded as a rudimentary or imperfectly developed penis, and, on the other hand, if the penis may be regarded as an exaggerated and hypertrophied clitoris—the one being, as it were, at the *minimum*, the other at the *maximum* of evolution—we can at once understand how every defect of development in the one case, and every excess of development in the other, must necessarily tend to the formation of an organ or structure intermediate between the penis and the clitoris.

It is by following out this principle of investigation, that we shall be enabled to explain very many, if not most, of the varieties of simple Hermaphrodism—the " hermaphrodisme sans exces" of continental writers.

For the purpose of facilitating and of giving precision to this enquiry, M. St. Hilaire divides the organs of generation in both sexes into three sections, viz: the external, the middle or central, and the deep-seated or internal. The external comprehends the penis and scrotum in the male, the clitoris and vulva in the female; the middle or central comprehends the prostate gland and vesiculæ seminales in the male, and the uterus in the female; and by the internal or deep-seated, he means the testicles with their appendages in the one sex, and the ovaries with their appendages in the other. This *trinary* division of the generative apparatus is much more important in M. St. Hilaire's opinion, than may on first consideration be imagined. It is not arbitrary, and adopted merely for the purposes of description. It is strictly and truly a natural division; for we shall find that each of the three sections is developed independently of the other two; and, therefore, that while one section may be formed perfect and entire, the others may be most imperfect and anomalous.

M. St. Hilaire has very ingeniously attributed this independence of formation to the anatomical arrangement of their vascular supplies. The three sections derive their blood from three different sources; and we shall find that the analogous or corresponding parts of both sexes are supplied by the same bloodvessels. Thus the ovaries and testicles are nourished by the spermatic arteries; the uterus and the prostate gland with the vesiculæ seminales by branches from the hypogastric; and the external organs by the pudic arteries.

This relation of the different parts of the sexual apparatus with the sources

* This circumstance explains an error which has been frequently committed in the history of monstrosities. It has been incorrectly said that they are much more common in the early female than in the early male fœtus.

of their vascular supplies is not accidental or subject to change. M. St. Hilaire believes it to be uniform and invariable, not only in the normal development of the organs, but also in monstrous irregularities. He has never found the organs of one of the three sections supplied by the arteries, which properly appertain to either of the others. For example, the uterus is never supplied by the spermatics, to the exclusion of branches from the hypogastrics; the testicles, on the other hand, are never supplied from the pudics, &c.

It is by keeping strictly in mind the two great laws or doctrines now adverted to—first, that in the young embryo the generative apparatus is without a determinate sex; and secondly, that the apparatus in each sex consists of three segments or divisions, which are in a great measure independent of each other in their development—that the philosophic anatomist has been enabled to solve some of the most puzzling enigmas of Hermaphrodism.

We do not mean to say that any light has been thrown on the mysterious question, what is the *cause* of those irregularities of development, which give rise to hermaphrodism. We cannot, indeed, expect to solve this question, until we have discovered what is the determining cause of the distinction of the sexes. Numerous very idle conjectures have at different times been proposed to explain this arcanum of nature; but it is almost unnecessary to say, that we know nothing certain on the subject. It is however worthy of mention, that there are some very curious facts in the natural history of some animals, which seem to throw a faint glimmering on the secret workings of this plastic Power, which generates, and evolves, and matures the wonders of animated creation. We shall very briefly allude to them.

Upwards of fifty years ago, our great countryman, John Hunter, published in the Philosophical Transactions an account of the animal, which is called by the farmers a "Free Martin." Our readers may not know what a Free Martin is. We shall therefore briefly explain its meaning. When a cow bears two calves at a litter, one of them being a male and the other appearing to be a female, this latter one is very generally an *imperfect* animal: in short, it is an Hermaphrodite, and is incapable, in after-life, of generation. Now this being an almost uniform occurrence in the cow tribe, some reasoners have been led to regard it as a proof, that there is a tendency in nature to the production of but one sex at the same time; and therefore that, when there are several fœtuses developed during one pregnancy, they have all more or less a disposition to assume the same sex. This however is altogether too loose and conjectural an idea to merit much attention.

The effect of removing the testes from the male and the ovaries from the female is, it is well known, to produce an animal neutral, as it were, and intermediate in appearance and character between the two sexes. The atrophy from age of these distinctive organs causes similar changes. Thus the females of some of the ruminantia, as the goat, deer, &c., sometimes acquire in their old age horns and antlers, although these appendages are, properly speaking, peculiar to the males of these animals. Analogous changes have been observed in other tribes of the mammifera. But it is especially in the class of birds, and more particularly in the members of the Gallinaceous family, that the approximation of the female to the male animal, under certain circumstances, is most conspicuous. When from age the hen ceases to lay eggs, or when from any cause, as disease of the ovaries, she is incapable of breeding, not only does her plumage often acquire all the characters of the cock bird, but often even a regular comb and wattles are formed. These changes have been observed to be more remarkable and decided in the gallinaceous than in perhaps any other tribe of birds.

From such facts as these now alluded to, it is not an unfair deduction to draw, that the decay of the energies or functions of one part of the generative apparatus has a marked tendency to approximate the animal to the condition and character of one of the opposite sex. The ovaries in the female, and the testicles in the male, are truly and strictly the *essential* organs of generation ; and *therefore* it is that an injury of these parts induces, in general, a decided change in the whole system of the body. Reasoning upon these data, our author has been led to form the ingenious conjecture, that in the production of simple Hermaphrodisme—'hermaphrodisme sans exces'—the cause or determining influence is exerted primarily on the ovaries or testicles of the embryo, and that the anomalies or irregularities of development in the other parts of the sexual apparatus are always consecutive to, and dependent upon the antecedent affection of these organs.

If this conjecture prove to be correct, a most important step in the etiology of Hermaphrodism would be attained ; for it would necessarily follow, that all the varieties of this strange irregularity of formation are attributable to a primary affection of the ovaries or of the testicles. The question then would come to be, can we form any rational conjecture as to the nature and cause of that primary affection ? M. St. Hilaire, while he admits that the subject is most mysterious, and that perhaps all our speculations amount at present to little better than ingenious guess-work, directs our attention to the possible effects, which atrophy or hypertrophy—in other words, an impeded or an exaggerated nutrition of the organs, from some inequalities in their vascular supplies—might have in the production of Hermaphrodism.

But it is time to quit the field of transcendental speculation, and to betake ourselves to the more humble task of simply narrating and describing some of the most common and the best-authenticated varieties of this malformation. We have already shewn that all the cases of Hermaphrodism may be arranged in one of two classes—according as the number of the generative organs *is*, or *is not* increased: and we have also shewn that these two classes may be conveniently subdivided into several orders or sections. The first class, that in which the number of the organs *is not* increased, Simple Hermaphrodism, [h. sans exces] comprises four orders—the masculine—the feminine, the neutral, and lastly the mixed. We shall briefly notice the most interesting characters of each of these in detail.

*Simple Masculine Hermaphrodism.* In examining a case of this variety, there may be considerable doubt during the life of the individual as to his proper sex, in consequence of the extreme degree of structural malformation of the external organs ; but all doubts are at once removed, if there is an opportunity of examining the body by dissection. The *essential* organs of the male generative apparatus will certainly be discovered : and the presence of these alone is always sufficient to determine the sex. The simplest form of masculine hermaphrodism is that condition of the male organs, where the penis and testicles are merely imperfectly developed; where the penis is unusually small, and where the testicles perhaps have not descended into the scrotum. Sir E. Home has described the case of a youth, 23 years of age, in whom the penis was of most infantine dimensions, and quite incapable of erection, and in whom the testicles were not larger than those of a year-old child. In this man, as indeed in almost all cases of a similar description, the whole system appeared to have sympathized with the *sub-masculine* condition of the generative apparatus. The body was weak and feminine, there was little hair on any part, the voice was thin and feeble, and the mammæ were unusually prominent.

Such is the first degree or simplest form of masculine hermaphrodism; the sexual organs are imperfectly developed, and the general constitution of the individual has acquired a character, neutral and, as it were, intermediate between those of the two sexes.

In the second form or degree of this variety, the malformation of the generative organs is much more serious, and their resemblance therefore to those of the female becomes much more striking. Not only is the penis always exceedingly small, but it is often imperfect at the same time. Thus in some cases the glans is imperforate; and in others the urethra is cleft on its lower surface along a greater or less extent, so that it has become, instead of a tube, a mere deep groove or open canal. In addition to these irregularities, the scrotum is frequently divided into two lateral parts by a deep fissure in the line of the raphe. In this manner the cleft scrotum takes on much of the external appearance of the vulva, with its labium pudendi on each side.

When the scrotal fissure is very deep, the resemblance to the female organs becomes strikingly great; for there is then formed a blind cavity or canal, which has in many cases been mistaken for an imperfect vagina.* To add to the puzzling character of such a case, it not unfrequently happens that, along with the malformations now described, the testicles have never descended through the inguinal rings.

In all cases of this second variety of masculine hermaphrodism, the evacuation of the urine is more or less difficult. Very generally the subjects of this unfortunate defect pass their urine 'à la maniere des femmes.' It is almost unnecessary to say that perfect coition must be impracticable.

---

* There was recently, in the wards of the Hôtel Dieu at Paris, an example of this variety of masculine hermaphrodism in a man 36 years of age. The scrotum, deeply divided by a central fissure, formed two lateral protuberances which contained the testicles. This fissure terminated in, or led into, a blind canal, which admitted the finger to the depth of about two inches. A probe could be passed to a considerably greater depth. To the upper part of the cleft scrotum was attached a miniature and imperfectly-formed penis. The urethra was open along all its lower surface, and the urine therefore dribbled away from between the two partitions of the scrotum.

This person it appears had had connexion with men. He had attempted, but in vain, to have intercourse with women.

From the description, given above, of the more aggravated form of masculine hermaphrodism, the reader will be prepared to expect that it is sometimes exceedingly difficult for a medical man (far less for a nurse or other unprofessional person) to determine the sex of a malformed infant at the time of birth. Numerous are the cases on record where even experienced anatomists have been obliged to confess their ignorance. Persons so afflicted have been not only baptized and educated, but have even subsequently been married, as females. Among other instances of this sort, there is one detailed at great length in the Memoirs of the Societé Med. d'Emulation for the year 1795, and another in the Bulletin de la Societé de Medicine for 1815. More frequently however, there are certain changes observable after the age of puberty, which cause suspicions as to the real sex of the individual; and our author adduces a multitude of cases, where the mistake was discovered at this period of life, and the party was re-baptized, and installed among the members of the ruder sex!

If from the study of Masculine we pass on to that of *Feminine Hermaphrodism*, we shall not fail to be struck with the very remarkable analogy, which may be shewn to exist between the irregularities of the male, and those of the female organs of generation, and to perceive at once the tendency, as it were, of the one sex to assume the characters of the other.

As the most frequent and distinguishing character of Masculine Hermaphrodism is the atrophied state of the penis, so the most remarkable anomaly of Feminine Hermaphrodism is the hypertrophied state of the clitoris. These two organs, it will be found, are strictly analogous to each other in structure, mode of development, and in the irregularities of their formation. Both consist of two spongy cavernous bodies, the corpora cavernosa, which, arising from the crus of the os pubis, were during early fœtal life distinct and separate from each other, and which afterwards coalesced in the mesial line, with a septum between their two lateral halves. The development of the urethra takes place independently of that of the corpora cavernosa; and in both sexes this canal occupies nearly the same relative position, although the great difference in the magnitude of the penis and of the clitoris in adult age is apt to withdraw the attention of the anatomist from this circumstance. When however the clitoris attains an unusual size, and especially when along with this anomaly the occasional, although certainly very rare, occurrence of its exhibiting a, more or less completely developed, urethral canal on its lower surface is present,* the resemblance between the generative organs

---

* Some writers have questioned the accuracy of the statement that the clitoris is ever perforated with a genuine canal. But all scepticism on this subject must be removed, after the perusal of the case of Marie Lefort, an account of which was published by M. Beclard in the Bulletin de la Faculté for 1815. The following is a brief abstract of the leading particulars. She was sixteen years of age when examined by M. Beclard. She is still alive, and M. St. Hilaire, a year or two ago, confirmed the exact accuracy of Beclard's description by a minute inspection of the woman.

" The prominence of the pubis is elongated as in the male subject, and covered with a quantity of hair. Immediately below the pubic protuberance is a body, about an inch and a half long, capable of enlargement during the state of erection. This body terminates in a glans, which however is imperforate, but is of the two sexes is too obvious to require any illustration. The resemblance becomes still greater if the vagina is at the same time imperfectly formed, being either exceedingly contracted, or perhaps quite imperforate. Such a state of parts approximates, it will be observed, very closely to the condition of the male organs in the second form of Masculine Hermaphrodism, described above. It may be supposed that, whenever the testicles have descended, we should be always able to determine the sex of a child, however malformed it may be; and most certainly we should, if in every case this circumstance could be ascertained beyond doubt. But, not to mention the frequently atrophied condition of these organs when the other parts of the generative apparatus are anomalous, we must remember that the ovaries occasionally descend through the inguinal apertures, and simulate most closely the form and general appearance of the testicles in the male.

In extreme cases therefore of Feminine Hermaphrodism,—when the clitoris is enormously developed, when it is cleft on its lower surface with an imperfect urethra from which the urine flows, when the vagina is unusually small and perhaps imperforate, when the labia are partially agglutinated, and when the ovaries have protruded at the inguinal apertures—the difficulty of determining the sex must be most puzzling indeed.

This difficulty becomes sometimes greater, as the person approaches the term of puberty; for the whole system is then apt to take on a sub-masculine character: the body and limbs are more muscular and hairy than in a female, the voice is more rough, the mammæ are less developed, and often too the mental and moral features exhibit none of the soft and graceful delicacy of the female mind.

We have alluded above to the mistakes which have been occasionally made as to the proper sex of a child at a period of birth, and during the years of

---

invested with a moveable prepuce. On its lower surface it is grooved or hollowed out, so as to form a canal (il est inferieurement creuseé d'un canal deprimé); and this canal is perforated at five different points along the median line. Below and behind this body there is a very imperfectly-formed vulva, and at the upper part of this vulva, immediately at the root of the clitoris, there is a rounded aperture, which admits the introduction of a sound.

The urine escapes partly by this opening and partly by the five little orifices, with which the urethra on the inferior surface of the clitoris is perforated (par les trous dont l'uretre est criblé dans sa portion clitoridienne). The catamenia escape by the same opening from which the urine flows, and perhaps also by the five orifices which we have mentioned:—at least they have been observed to be moistened with the sanguineous discharge.

The case therefore of Marie Lafort may be justly considered as affording an indubitable instance of the urethra, incomplete indeed, being developed along the inferior surface of the clitoris, as it normally exists in the male penis.

This rare malformation is an admirable illustration of the 'Unity of Composition' of the analogous or corresponding structures of the male and of the female generative apparatus. 'If then,' says M. St. Hilaire, 'the penis and clitoris, organs essentially analogous to each other, are formed of the same anatomical elements, if in short they are but different degrees of evolution of the same organic tissues, it is not more wonderful that the clitoris should occasionally be developed up to the complex condition of the penis, than that the latter should descend to the rudimentary state of the clitoris."

childhood and early youth; and we have mentioned that male children have been baptized and educated as girls, and that it was not until after puberty, when the growth of the sexual passions and other circumstances took place, that the awkward mistake has been discovered. Instances are also on record where female children have been christened as boys, and have been educated as such.

Our author has described several cases of this description, and to his work we therefore refer those readers who wish to know the particulars. We shall only allude to the case of a young person, who, after having been educated as a boy and afterwards admitted as a canon in a monastery, subsequently became pregnant and gave birth to a living child!! It was upon this person that the line—

Mas mulier, monachus, mundi mirabile monstrum, was written.

From the preceding remarks on masculine and on feminine hermaphrodism, we hope that our readers may be enabled to form an accurate notion respecting these two varieties of sexual malformation. We have shewn that the malformation may be so great, that it is almost quite impossible in some cases to determine the sex of the unfortunate individual even after puberty;* and, notwithstanding, this extreme deviation, that all the anomalies of structure may be accounted for on the principle of the strict and most intimate resemblance in development of the external organs of generation, or organs of *copulation*, in the male and in the female. We have not yet alluded to the condition of the internal organs, or those of *reproduction;* and the reason of our silence has been, that these parts are not necessarily malformed in the two varieties of simple Hermaphrodism, of which we have hitherto spoken. The irregularity of formation in them is limited to the external organs; and hence the dissection of any case of genuine masculine or feminine Hermaphrodism will at once determine the order or variety to which it belongs.

---

* On perusing lately the interesting literary correspondence of Baron Grimm, we met with the following droll case. Anne Grandjean, born at Grenoble, was baptized as a girl, and educated as such until she was fourteen years of age. From the change in her feelings and conduct at that time, doubts as to her proper sex arose. The Confessor of the family was consulted, and he decided that the child should be dressed and treated as a boy. Some years afterwards *he* made love to a young lady, but for some reason or another, the acquaintance was broken off. A second attachment however proved more successful, and he was married.

The couple lived together for two or three years at Lyons. It so happened that his first flame came to reside there, and became acquainted with Madame Grandjean. The two ladies appear to have conversed about family topics; for the issue of all was, that the wife declared that her husband was not "un veritable homme." She applied to the church for redress; and at length poor M. Grandjean was condemned to be whipped and expelled from Lyons, "en qualité de profanateur du sacrament de mariage." He appealed however to the Parliament of Paris, which revoked the sentence of the tribunal at Lyons, declared his marriage null, and at the same time ordained that he should resume the name and garb of a woman!! Well might Baron Grimm remark, "que cette affaire n'aurait jamais du faire un sujet de proces public dans un siecle eclairé."

If, along with malformation of the external organs, there is found an anomalous state of any of the internal, the case is still more complicated than any we have yet considered, and it is to be referred to one of the other two varieties of simple hermaphrodism—the *neutral* or the *mixed*.

The essential and characteristic feature of Neutral hermaphrodism is, that all or at least most of the organs of generation, internal as well as external, are strictly neither male nor female, but are intermediate between those of the two sexes, and partaking, as it were, of the attributes of both.

Hitherto no very authentic example of this variety of hermaphrodism has been recorded to have been observed in the human subject; and, even in those cases which have occurred in some of the lower animals, the confusion of development did not equally affect all the organs alike; the external, it would seem, appertaining in most of them decidedly to the female sex. We shall briefly notice three of the most remarkable cases of Neutral hermaphrodism. They are recorded by Haller, Hunter, and Sir E. Home.

The case observed by Sir E. Home occurred in a dog. The external organs consisted of a vulva tolerably well formed, and of a large clitoris, or a small imperforated penis, beneath which there was an aperture leading to the urethra. Such a state of parts appeared to indicate most essentially the female sex; and of such was the animal considered, until the internal apparatus was examined. This was found to consist of only an elongated ligamentous substance—regarded by Home as an imperforated vagina—and of two slender impervious cords terminating in two bodies, whose structure and general appearance was so equivocal, that it was quite impossible to decide whether they were imperfect ovaries or testicles. They occupied indeed the situation of the ovaries; but still their characters were such as to lead Sir Everard to consider them rather as testicles. On the whole however, we must regard the preceding case as one rather of extremely irregular and defective development of the female organs, than of strictly Neutral hermaphrodism.

In the animal of the goat tribe observed by Haller, there was either a large clitoris, or small penis, according to the view taken of the case. Externally it was perhaps more like to the former organ; but when attentively examined, Haller stated that it resembled rather the penis of the male: its two corpora cavernosa being very distinct and furnished with very obvious muscles. Beneath this organ, there was a fissure similar to a very narrow vulva; a probe might be introduced along it either into the bladder, or into a vagina-sort of canal, placed between the rectum and bladder, and communicating with the urethra by a large aperture. This canal was found on dissection to bifurcate into two tubes. These two tubes, resembling rather the Fallopian tubes or even the cornua of the uterus than proper vasa deferentia, terminated in bodies which, although occupying the usual site of the ovaries, looked like testicles. Around the extremitiy of the urethra, at its junction with the bladder, there were observed traces of rudimentary vesiculæ seminales.

The neutral hermaphrodite described by Hunter was very similar, in most respects, to the preceding one. It occurred in an ox. The external parts belonged rather to the type of the female animal. The vagina was very imperfect; the uterus was impervious; and the two ovaries had much more the appearance of testicles, and in truth Hunter regarded them as such.

There were also rudiments of vesiculæ seminales around the neck of the bladder in this animal.

The last form or variety of simple hermaphrodism is what has been denominated *mixed hermaphrodism*, from the circumstance of some of the organs of generation being to all appearance male, while others appear equally to be truly female.

There is thus, as it were, an admixture of the two sexes, occurring in different parts of the same apparatus. Authors have described two groupes of mixed hermaphrodism. In *one*, the external organs being more or less irregular, some of the internal organs are masculine, while others are of a feminine character. Thus there may be testicles coexistent with a uterus, or, on the other hand, there may be ovaries coexistent with the prostate gland and vesiculæ seminales.* In the *second* groupe, one lateral half of the apparatus appears to appertain to the male sex, while the other lateral half appears to belong to the female sex.

We shall briefly allude to a few cases, which have been supposed to exemplify these rare anomalies.

As an instance of the *first* groupe of mixed hermaphrodism, Akerman published at Jena in 1805 the following account of the state of the generative organs in a new-born child.

Externally there was a large clitoris, or diminutive imperforated penis, grooved on its under surface; beneath this body, was a vulva, in the labia of which were discovered testicles. This vulva, or, if you choose, this scrotal fissure led by one passage to the urinary bladder, and by another to a thin globular uterus, from which there arose two narrow tubes, analogous to vasa deferentia, and terminating in the two testicles. The structure of the testicle was normal, and, as already observed, they had passed through the inguinal apertures.

The celebrated anatomist, Mayer, has recorded a somewhat similar case, which he detected in a young fœtus. There was a bicorned uterus, and a vagina, associated with testicles, vasa deferentia, and rudiments of vesiculæ seminales. The urinary bladder was affected with extro-version, and received at its fundus the orifice of the vagina.

M. St. Hilaire has described several examples of this mixed hermaphrodism in the lower animals. We must refer those who wish for particulars to his work.

The *second* groupe of mixed hermaphrodism consists, as we have explained above, in the right lateral half of the generative apparatus assuming the

---

* The trinary division of the generative organs suggested by our author, and alluded to by us in a former part of this article, is used with much advantage in describing this variety of mixed hermaphrodism. It will be remembered that he divides these organs into the external, the middle, and the deep or internal: the middle consisting of the prostate gland and vesiculæ seminales in the male, and of the uterus in the female; and the internal organs consisting of the testicle and its appendages in the male, and of the ovaries and its appendages in the female. Applying therefore this language to the description of the first groupe of mixed hermaphrodism, we should say that the middle organs may be masculine, while the internal organs are feminine, or vice versâ; the external organs being, in both sets of cases, generally more or less irregular at the same time.

masculine characters, and the left assuming the feminine; or vice versa. This is a very rare form of monstrosity in the higher animals; but it is infinitely more common in some of the lower tribes, especially in Fishes and in the Articulata. This fact, the result of undoubted observation, is strictly in accordance with, and affords, in our author's opinion, a most beautiful illustration of the agency of Centripetal development. "If," says he, "it is true that lateral hermaphrodism may be explained on the principle, that the two lateral halves of the sexual apparatus are evolved and formed distinct from and independent of each other, it is evident that the production of this anomaly must be more apt to occur, and therefore be more frequent, just in proportion as the development of these halves is more tardy, and their coalescence in the mesial plane is more slow and more incomplete. Now in man, and in those animals which approach him in organization, each lateral demi-apparatus speedily unite together on the mesial line, and their fusion or coalescence is subsequently so complete, that no trace of their former duality is found. In the inferior mammiferous animals, the mesial coalescence is less complete; for in them we find a double uterus, and sometimes also a double vagina."

If we descend still lower in the scale of organization, we find that the right and left lateral halves remain disunited throughout, and discharge their contents separately at the anus, or into a common cloaca. Hence we are furnished with a very simple and very satisfactory explanation of the frequency of lateral hermaphrodism in the lower tribes of animals; and of its extreme rarity in man and the higher animals. Whenever it does take place in these, it is attributable to an arrest of the progressive development of the sexual apparatus.

The following example of this rare variety of Hermaphrodism is narrated by Marcet in the Memoirs of the Academy of Dijon, and has been inserted in most of the recent works on the malformations of the generative organs. The person, who had always passed as a male, died at the age of 17.

There was a large clitoris—or diminutive imperforate penis—provided with a distinct glans and prepuce, and consisting (as ascertained by dissection), of two well-developed corpora cavernosa. Immediately beneath this organ, there was a considerable fissure formed by two folds of the skin, which represented tolerably well the labia of the vulva, and within which were nymphæ-like bodies. These were separated superiorly by the meatus urinarius, inferiorly by a narrow orifice, which seemed to be the opening of a vagina. Within the left labium of the vulva—or lip of the scrotal division —was felt most distinctly a testicle. There was not one in the right labium; but, when the abdomen was firmly pressed, an oval substance protruded through the inguinal ring, and this receded, when the pressure was removed.

On dissection, the substance within the left labium was found to be "un vrai testicule pourvu de ses vaisseaux, et d'un canal deferent:" the vas deferens passed through its corresponding inguinal ring, and proceeded on to a vesicula seminalis on the left side of the neck of the bladder. From this description it appears that the left side of the generative apparatus was most strictly and truly *masculine*. Not so however with the right *side*;—for, with the exception of some imperfect rudiments of a vesicula seminalis and of a vas *feminine*, it was essentially *feminine* in its arrangement. The small oval substance which had protruded at the right inguinal ring, and which had been supposed during life to be a testicle, was found to be a diminutive uterus, contained in a cyst. It was an inch and a half long, of a firm texture, and, when cut across, proved to contain a small ovoid cavity. From its upper part there arose a genuine fallopian tube, which proceeded on to an ovary, normal as to colour, form and volume, and " dont l'existence est par consequent aussi authentique que celle du testicule gauche."

A case, somewhat similar to the preceding one, was discovered in the body of a subject examined at the Hôtel Dieu in 1754. The canal of the urethra, which terminated externally an inch from the extremity of the penis, was very loosely connected to the corpora cavernosa by mere cellular substance. Beneath this body was a fissure, the two sides of which looked like the two halves of a fissured scrotum. In the right one was felt a testicle provided with its spermatic cord. Dissection proved that, besides a distinct vas deferens, there was a vesicula seminalis on the right side of the bladder, and that this vesicula communicated not only with the urethra, but also with a small oval uterus. This uterus was provided with a broad and also with a round ligament, which proceeded on through the left inguinal aperture, and was there lost in the cellular substance of the part.

Our limits prevent us from describing any more cases of lateral Hermaphrodism in man and the higher animals. But of all the Vertebrated animals, there is no class in which this malformation has been so frequently found as in Fishes; and it would seem that, in them, so far from preventing the accomplishment of the sexual functions, this very abnormal arrangement of the two lateral halves of the apparatus enables the animal to act both as a male and a female—or, in other words, to form and to excrete the spermatic fluid, and also the ova at the same time.

Many works on comparative anatomy describe cases, in which there has been found in the abdomen of a variety of fishes a mass of ova on the one side, and a well-developed milt on the other. Indeed the complete separation and independence of the two lateral halves of the generative organs in fishes, and the simplicity of their reproductive functions, seem quite to establish the possibility of one animal impregnating its own ova with its proper seminal fluid—the realisation of *genuine Hermaphrodism.*

We have now concluded our imperfect description of the different varieties of simple Hermaphrodism—the " H. sans exces,"—in which there is no abnormal augmentation of the number of the generative organs, but only a malformation or irregularity of their development. We have attempted to shew that they are most conveniently arranged into four groupes—the *masculine,* in which the external organs only of generation are more or less malformed, and approach in appearance and configuration to the female apparatus; the *feminine,* in which the opposite condition of parts exists; the *neutral,* in which all the organs, internal middle and external, are so malformed, that they cannot be truly said to appertain to either sex, but rather to be confusedly intermediate between the two; and lastly the *mixed,* in which, while the external organs are almost always irregularly and imperfectly formed, the middle organs seem to belong to one sex, and the internal organs to another. Although the limits between these different groupes are not uniformly distinct and well marked, the classification will be found very convenient for arrangement and for scientific description.

The other grand division of Hermaphrodism—in which there is not only a malformation, but also an actual increase of number, in the organs of generation,—either from some of the male being superadded to a female apparatus, or from some of the female being superadded to a male apparatus—has been denominated Complex Hermaphrodism, or " H. avec exces."

It has been proved that many of the anomalies of Simple Hermaphrodism are explicable, more or less satisfactorily, on some discovered law of Embryogenic development. Not so with the etiology of Complex Hermaphrodism. In truth, we cannot form any rational conjecture as to the modus operandi of the formative power, in giving rise to the production of supernumerary organs.

Leaving therefore this dark and mysterious subject, we shall satisfy ourselves with merely recording two or three of the most memorable and best authenticated examples of the Hermaphrodism " avec exces."

The following is one, wherein one or two feminine organs were superadded to a male apparatus. It is recorded by Petit in the History of the French Academy of Sciences for the year 1720. The man was 22 years of age, and died from the effects of a wound. The external parts were most distinctly male; the penis was quite well formed, but the scrotum was void of testicles. These glands however were found on dissection to be situated in the loins, and were provided with very obvious epididymes and vasa deferentia. The prostate gland and vesiculæ seminales also were normally formed. They opened as usual into the urethra; but, in addition to their orifices in the prostatic portion of this canal, there was another, which proved to be the opening of a uterus attached to the neck of the urinary bladder. From either side of this uterus there arose a Fallopian tube, which proceeded on to the epididymes, but it had no fimbriated extremities. Professor Mayer detected a similar excess of parts in a six-month fœtus: the male organs were complete, and in addition to these there was a uterus, which opened into the urethra near the neck of the bladder. This strange anomaly of formation has been discovered in several of the lower mammiferous animals also.

Such cases are grouped under the title of Masculine Complex Hermaphrodism—the apparatus being decidedly male, with the super-addition of certain female organs. The groupe opposed to this is denominated Feminine Complex Hermaphrodism and is exemplified in the following instances.

Beclard has described such a case, in which the external organs exhibited the appearance of the second degree of simple Masculine Hermaphrodism. There was a diminutive imperforated penis, or an exaggerated clitoris; a vulva, or fissured scrotum, in the lips of which were to be felt the testicles. But on examining the internal organs, every thing indicated a female apparatus. There was a vagina, a uterus with its fallopian tubes, and two ovaries. The vasa deferentia leading from the testicles passed on to the sides of the uterus, and terminated where the round ligaments are usually situated.

The case minutely reported by M. Bouillaud about two years ago, and inserted in the Number of this Journal for July 1833, belongs to the present groupe. The person, aged 62, had lived and been married as a man. On dissection it was found that there was no appearance of scrotum or testicles, the raphé extending from the anus to the imperfect penis or clitoris. This organ was of considerable size, but the canal of the urethra terminated on its inferior surface behind the glans, as in the first degree of hypospadias. All the internal organs were decidedly female; the uterus however terminated in the urethra near to the neck of the bladder. In addition to these parts, there was a well-formed prostate gland in its usual situation.

A third groupe of Complex Hermaphrodism comprehends those very rare cases, in which the male and the female apparatus—more or less complete—exist together in nearly the same degree of development. Schrell, a German anatomist has described a case, in which he found that, in addition to a "veritable penis," and also to normally-formed testicles provided with vasa deferentia, there was a small vulva with its labia and nymphæ, a vagina leading to a rudimentary uterus, provided with Fallopian tubes and with imperfectly developed ovaries. In 1826, Dr. Harlan of Philadelphia published a minute account of an hermaphrodite ourang-outang, in which the male and the female generative apparatus were, in a nearly equal degree, co-existent. The description is full of interest, and will no doubt be studied with attention by all who take an interest in the science of Teratology. Mascagni also, the celebrated anatomist of Italy, has narrated an extraordinary case, which he met with in a bull. This animal had actually been employed for the purposes of breeding. The external organs were well formed, and every thing indicated a normal state of parts; but on dissection there was found a vagina which opened into the urethra between the orifices of the seminal ducts, a uterus, and one ovary, situated near to the left testicle. The author himself closes his narration in the following words: "Si riunivano le parti maschili della generazione in tutto o per tutto perfette, e come maschio dove aver generato: vi si riunivano quelle della femina, ad eccezione delle parti esterne che mancavano in totalità."

In concluding our brief description of the different kinds of Hermaphrodism, the reader, we trust, cannot fail to have perceived the harmony of most of the phenomena, presented by the examination of these malformations, with the general laws of Organology which we have endeavoured to illustrate in the early pages of this article.

He will now understand that most of those congenital irregularities of structure, which have been called Monstrosities, are easily and rationally explicable, without having recourse to the interposition of evil spirits or witches, as our good forefathers supposed, or even to the agency of any sportive freaks of Nature, as many good naturalists have told us. Aristotle denominated them "errors of creation;" Pliny mentions them in these words, "ludibria sibi, nobis miracula ingeniosa fecit natura;" and even in modern works they are often alluded to as "blind and chance structures," "inexplicable wonders" "bizarre and sportive productions" and so forth. Such language can never be properly applicable to any of the works of Nature. We may be satisfied that there is no caprice, or waywardness, or inconstancy in the laws which regulate the formation and development of these; and if at any time there appears to be such, we may rest assured that it is, because our ignorance cannot understand, and our feebleness cannot comprehend them. It is certainly a grand reflection that even the very irregularities or errors, if we may use the term, of Creation afford to the philosophic observer convincing proofs of that harmony of operation which moves all, and of that unity of design which has planned and formed and established all. Does not this thought lead us, almost unconsciously, to the conclusion that every part of Nature proclaims the impress of one great Mind, and teach us to know that there is but one mighty Architect, alone and for ever the same.

